# The mismatch repair and meiotic recombination endonuclease Mlh1-Mlh3 is activated by polymer formation and can cleave DNA substrates in trans

**DOI:** 10.1371/journal.pbio.2001164

**Published:** 2017-04-28

**Authors:** Carol M. Manhart, Xiaodan Ni, Martin A. White, Joaquin Ortega, Jennifer A. Surtees, Eric Alani

**Affiliations:** 1 Department of Molecular Biology and Genetics, Cornell University, Ithaca, New York, United States of America; 2 Department of Biochemistry and Biomedical Sciences, McMaster University, Hamilton, Ontario, Canada; 3 Department of Molecular and Cellular Biology, Harvard University, Cambridge, Massachusetts, United States of America; 4 Department of Biochemistry, University at Buffalo, State University of New York, Buffalo, New York, United States of America; Rockefeller University, United States of America

## Abstract

Crossing over between homologs is initiated in meiotic prophase by the formation of DNA double-strand breaks that occur throughout the genome. In the major interference-responsive crossover pathway in baker’s yeast, these breaks are resected to form 3' single-strand tails that participate in a homology search, ultimately forming double Holliday junctions (dHJs) that primarily include both homologs. These dHJs are resolved by endonuclease activity to form exclusively crossovers, which are critical for proper homolog segregation in Meiosis I. Recent genetic, biochemical, and molecular studies in yeast are consistent with the hypothesis of Mlh1-Mlh3 DNA mismatch repair complex acting as the major endonuclease activity that resolves dHJs into crossovers. However, the mechanism by which the Mlh1-Mlh3 endonuclease is activated is unknown. Here, we provide evidence that Mlh1-Mlh3 does not behave like a structure-specific endonuclease but forms polymers required to generate nicks in DNA. This conclusion is supported by DNA binding studies performed with different-sized substrates that contain or lack polymerization barriers and endonuclease assays performed with varying ratios of endonuclease-deficient and endonuclease-proficient Mlh1-Mlh3. In addition, Mlh1-Mlh3 can generate religatable double-strand breaks and form an active nucleoprotein complex that can nick DNA substrates in trans. Together these observations argue that Mlh1-Mlh3 may not act like a canonical, RuvC-like Holliday junction resolvase and support a novel model in which Mlh1-Mlh3 is loaded onto DNA to form an activated polymer that cleaves DNA.

## Author summary

In sexually reproducing organisms, crossing over between homologous chromosomes in meiosis creates physical linkages required to segregate chromosomes into haploid gametes. In baker’s yeast, which utilizes meiotic recombination pathways conserved in mice and humans, the majority of meiotic crossovers are initiated through the formation of a branched DNA intermediate, which is stabilized by the Msh4-Msh5 complex. This DNA intermediate is further processed to form a structure (a double Holliday junction), which requires the endonuclease activity of the Mlh1-Mlh3 DNA mismatch repair factor to be resolved exclusively into a crossover product. Current meiotic recombination models invoke the use of structure-specific enzymes that symmetrically cleave single Holliday junctions. In this study, we provide evidence that the yeast Mlh1-Mlh3 complex is unlikely to act as a structure-specific enzyme. Furthermore, we showed that Mlh1-Mlh3’s endonuclease activity is dependent upon its ability to form a polymer on DNA and suggest that it is capable of cleaving DNA that is captured in an active complex. Together, our biochemical observations support a novel model involving regulated polymerization of Mlh1-Mlh3 for its cleavage function, potentially in meiotic crossovers or in mismatch repair.

## Introduction

Mismatch repair (MMR) acts during DNA replication to remove DNA polymerase misincorporations such as single-nucleotide base–base mismatches and insertion or deletion loops that result from polymerase slippage events. In *Escherichia coli*, DNA mismatches are initially recognized by MutS, which recruits MutL in an ATP-dependent reaction to activate MutH. MutH functions as a latent endonuclease that nicks the newly replicated and undermethylated DNA strand up to several kilobases away from the mismatch. The resultant nick, 5′ or 3′ to the mismatch, directs excision and resynthesis steps that remove the misincorporated DNA [1].

MMR is highly conserved across organisms. In baker’s yeast, mice, and humans, MutS homologs (MSHs) form heterodimers Msh2-Msh6 and Msh2-Msh3, which recognize and bind with overlapping specificities to base–base mismatches and insertion or deletion loops. These MSH complexes primarily interact with the MutL homolog (MLH) complex Mlh1-Pms1 (MLH1-PMS2 in humans), which nicks the newly replicated strand to promote excision and resynthesis in steps coordinated with a yet-to-be-understood strand discrimination mechanism [1]. A minor MMR pathway has been identified in baker’s yeast in which Mlh1-Mlh3 interacts with Mlh1-Pms1 to repair a subset of DNA mismatches that are recognized by Msh2-Msh3 [2–4].

A subset of MSH and MLH protein complexes act during yeast meiosis to promote the major class of crossovers (COs) that form between homologs. In *Saccharomyces cerevisiae*, meiotic recombination is initiated by the formation of ~200 programmed double-strand DNA breaks (DSBs) that appear throughout the genome. During prophase I, DSBs are resected to form 3′ single-strand tails, which are directed to invade and pair with the unbroken homolog. About half of these invasions are stabilized by a family of ZMM proteins (Zip1-4, Mer3, and Msh4-Msh5) to form single-end invasion (SEI) intermediates. The SEIs are further processed through synthesis and ligation steps to form double Holliday junction (dHJ) intermediates, which are subsequently resolved exclusively into COs [5–7]. Invasion intermediates that are not protected by ZMM proteins can also mature into dHJs but are not sensitive to interference and are resolved into both COs and noncrossovers (NCOs). Work in *Sordaria*, mouse spermatocytes, and human oocytes suggested that Msh4 foci (presumably bound to Msh5) form in leptotene at all DSB sites [8–11]. As meiosis progresses, the number of Msh4 foci decrease. When the cell enters pachytene, Msh4 foci were found to colocalize with Mlh1 foci at sites that ultimately form COs [12].

How does the Mlh1-Mlh3 endonuclease act to resolve dHJs to form crossovers? Genetic and molecular work performed in baker’s yeast showed that dHJs stabilized by ZMM proteins are resolved through the actions of factors that include the Mlh1-Mlh3 endonuclease, Sgs1-Top3-Rmi1, and the exonuclease-independent functions of Exo1 [13,14]. Resolution of dHJs in this pathway is biased to cut the two Holliday junctions (HJs) in opposite orientations to produce exclusively COs [15–23]. Interestingly, the HJ resolvases Mus81-Mms4, Slx1-Slx4, and Yen1 can compensate for the absence of Mlh1-Mlh3, but dHJs are processed through an interference-nonresponsive pathway that randomly resolves dHJs into COs and NCOs. These structure-selective HJ resolvases, which can bind to and cleave synthetic HJs and other branched structures at specific sites in vitro, act independently of the ZMM proteins and in the presence of Mlh3 account for only a minority of crossover events [18] (reviewed in [24]). Mus81-Mms4 and Yen1 are regulated by phosphorylation in a cell cycle–dependent manner [25].

Previously, we showed that yeast Mlh1-Mlh3 is an endonuclease whose activity is stimulated on plasmid substrates by yeast mismatch repair factor Msh2-Msh3 but not the replication processivity clamp/clamp-loader (PCNA/RFC), which stimulates Mlh1-Pms1 [26,27]. Mlh1-Mlh3 displays binding preference for oligonucleotide substrates containing mismatches and branched DNA structures, particularly HJs [26,27], but does not display endonuclease activity on these oligonucleotide substrates, even in the presence of Msh2-Msh3. This behavior is significantly different from the biochemical activities of the well-characterized HJ resolvases Mus81-Mms4, Slx1-Slx4, and Yen1, which cleave such substrates (reviewed in [7,24]), but is consistent with activities seen for the MMR endonuclease Mlh1-Pms1 in yeast or MLH1-PMS2 in humans ([28,29]; see below). In vivo analysis of MMR factors in *S*. *cerevisiae* suggests that multiple Mlh1-Pms1 complexes bind to a DNA mismatch [30]. In vitro, yeast Mlh1-Pms1 exhibits high affinity and cooperative binding to large DNA substrates [31], and single-molecule studies showed that yeast Mlh1-Mlh3 and Mlh1-Pms1 have similar diffusion properties on duplex DNA [32,33]. These observations indicate that DNA-binding properties are conserved among MLH family members; in support of this idea, work in *E*. *coli* suggested that multiple MutL proteins interact with a MutS-bound mismatch substrate [34].

Because MLH proteins display similar DNA binding properties that appear critical for their roles in MMR, we hypothesize that such properties are utilized by Mlh1-Mlh3 in meiosis to resolve dHJs into COs. Here we show that Mlh1-Mlh3 displays an endonuclease activity on large duplex DNA substrates; DNA binding, wild type and mutant protein complex mixing experiments, and electron microscopy assays indicate that Mlh1-Mlh3 polymer formation is required for this activity. We also observe that Mlh1-Mlh3 is capable of making DSBs in a concerted manner that are religatable. Finally, we observe that Mlh1-Mlh3 is capable of cleaving DNA substrates in trans. These data support a novel model in which a polymer of Mlh1-Mlh3, positioned and directed by other meiotic recombination proteins, may cleave recombination intermediates to form COs. These properties distinguish Mlh1-Mlh3 from structure-selective endonucleases found in other dHJ cleavage pathways.

## Results

### Mlh1-Mlh3 does not have the hallmarks of a structure-selective endonuclease

**Fig 1:**
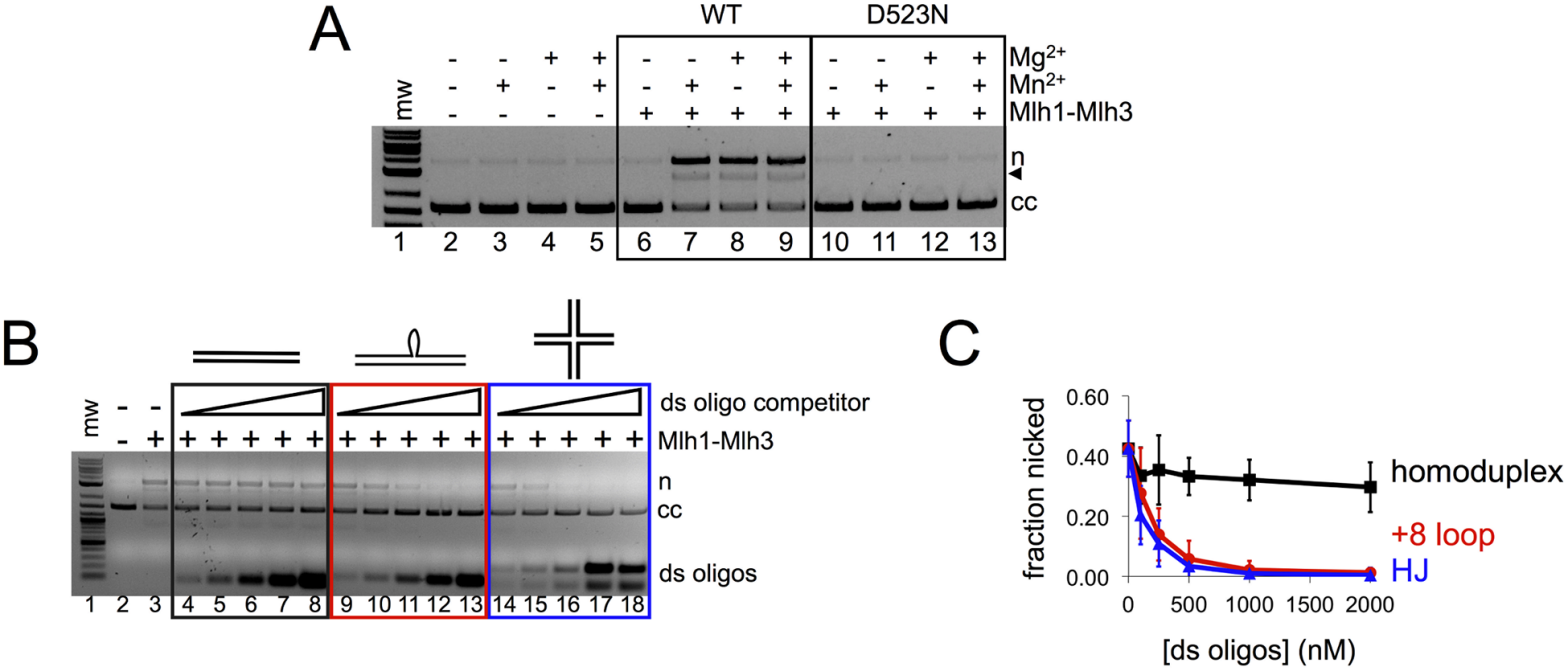
Mlh1-Mlh3 binding to mismatched and Holliday junction substrates inhibit its endonuclease activity. In native agarose gels, migration of nicked product (n), linear product (black triangle), and closed circular substrate (cc) are indicated. All endonuclease reactions were carried out for 60 min, then stopped by addition of sodium dodecyl sulfate (SDS), ethylenediaminetetraacetic acid (EDTA), and ProteinaseK as described in the Materials and methods. (A) Endonuclease activity was performed with supercoiled pUC18 as described in the Materials and methods. Where + Mg^2+^ or + Mn^2+^ is indicated, 1 mM MgCl_2_ or MnSO_4_ was added. Where both Mg^2+^ and Mn^2+^ are indicated, 0.5 mM of each was included. Where + Mlh1-Mlh3 is indicated, 300 nM wild type or Mlh1-mlh3D523N was added. (B) Mlh1-Mlh3 endonuclease activity on a 2.7 kb circular DNA substrate is inhibited by preincubating Mlh1-Mlh3 with oligonucleotide substrates. Mlh1-Mlh3 (100 nM) was preincubated with increasing amounts of ~50 bp double-stranded oligonucleotide substrates for 15 min at 30°C (0–2,000 nM): either homoduplex (0 μM, 10 μM, 25 μM, 50 μM, 100 μM, or 200 μM, expressed as total nucleotide concentration), +8 loop (0 μM, 10 μM, 25 μM, 50 μM, 100 μM, or 200 μM, expressed as total nucleotide concentration), or 30 bp armed Holliday junction (0 μM, 24 μM, 60 μM, 120 μM, 240 μM, or 480 μM, expressed as total nucleotide concentration). After the preincubation step, reactions were challenged with ~18 μM (expressed as total nucleotide concentration) 2.7 kb circular substrate and incubated by conditions described for endonuclease assays in the Materials and methods and analyzed by agarose gel. All lanes contain 1 mM Mg^2+^. (C) Average of quantification of plasmid nicked for four separate experiments from B; error bars represent standard deviation.

Mlh1-Mlh3 is active on plasmid substrates in the presence of divalent magnesium or manganese, with manganese giving slightly greater nicking activity ([26,27]; Fig 1A). We did not observe endonuclease activity with either metal using purified Mlh1-mlh3D523N, a complex bearing a mutation that disrupts a conserved residue seen in the MutL family endonucleases ([19,26,27]; Fig 1A).

Curiously, Mlh1-Mlh3 shows preferential binding (Fig 1B and 1C) to a variety of branched DNA structures such as HJs and insertion or deletion loop mismatch DNA substrates but does not cleave them ([26,27]). In addition, when a cruciform was incorporated into a plasmid substrate to mimic a HJ intermediate, specific nicking at the cruciform was not observed [27]. These observations suggested to us that Mlh1-Mlh3 does not display structure-specific nuclease activities on HJ substrates, as was seen for RuvC, Yen1, and Mus81-Mms4. To further test this, we performed the three experiments described below.

First, we observed that incorporating a +8 loop mismatch into a plasmid substrate had an inhibitory effect on Mlh1-Mlh3 endonuclease activity and did not localize its endonuclease activity to sites near the mismatch (see details in the next section). Second, we performed an oligonucleotide competition endonuclease assay in which Mlh1-Mlh3 was incubated with a circular plasmid substrate in the presence of homoduplex, HJ, and +8 loop–containing oligonucleotides. As shown in Fig 1B and 1C, HJ and +8 loop–containing oligonucleotide substrates acted as very strong competitors relative to homoduplex DNA, which was an ineffective competing substrate. The differences in competition efficiency among the oligonucleotide substrates were much greater than Mlh1-Mlh3’s DNA-binding affinities for these substrates—the K_d_ values are in the nanomolar range for all substrates [26,27]. Finally, we tested whether Mlh1-Mlh3 activity is sensitive to phosphorylation. In meiosis, Mus81-Mms4 and Yen1 endonuclease activities are regulated by their phosphorylation state. Phosphorylation of Mus81-Mms4 by Cdc5 has been shown to hyperactivate the endonuclease, while Yen1 is inhibited by phosphorylation [25]. Based on Mlh1-Mlh3’s identity as an MMR factor, its activity is not expected to be sensitive to phosphorylation; there is no evidence in the literature that MLH endonuclease activity is regulated by phosphorylation. Nevertheless, we treated Mlh1-Mlh3 with either lambda protein phosphatase or CDK1-cyclinB, which has a recognition motif in Mlh3. We then compared the protein’s endonuclease activity to controls not treated with a phosphatase or kinase (S1 Fig). We did not observe an effect on endonuclease activity when Mlh1-Mlh3 was treated with a phosphatase or kinase, suggesting that it is unlikely to be sensitive to these modifications. Together, our data argue against Mlh1-Mlh3 acting according to archetypes set by canonical HJ resolvases, and HJs and mismatched substrates are unlikely to be the preferred in vivo substrates for the Mlh1-Mlh3 endonuclease.

### Mlh1-Mlh3 forms a polymer, which activates its endonuclease function

Previously we showed in electrophoretic mobility shift assays (EMSA) that a specific Mlh1-Mlh3-DNA gel shift could only be detected in a narrow range of Mlh1-Mlh3 concentrations that are suboptimal for nuclease activity. At higher concentrations, a more robust nuclease activity was seen, but in EMSA assays performed at these concentrations, Mlh1-Mlh3-DNA complexes do not enter the gel [26,27]. This observation and the lack of endonuclease activity on oligonucleotide substrates suggested to us that multiple Mlh1-Mlh3 heterodimers are required to activate its endonuclease function. The more thoroughly studied yeast Mlh1-Pms1 complex was shown to bind larger DNA substrates with higher affinity than smaller DNA substrates [31]. Hall et al. hypothesized that this behavior is suggestive of cooperative binding, which necessitates the presence of multiple protein complexes [31]. They supported this biochemical observation using atomic force microscopy, where they observed long tracts of protein bound to plasmid DNA. Consistent with the in vitro data, multiple (~15) Mlh1-Pms1 molecules are recruited to mismatch sites in vivo [30]. These observations encouraged us to test whether disruptions to DNA substrates inhibit Mlh1-Mlh3’s endonuclease activity.

**Fig 2:**
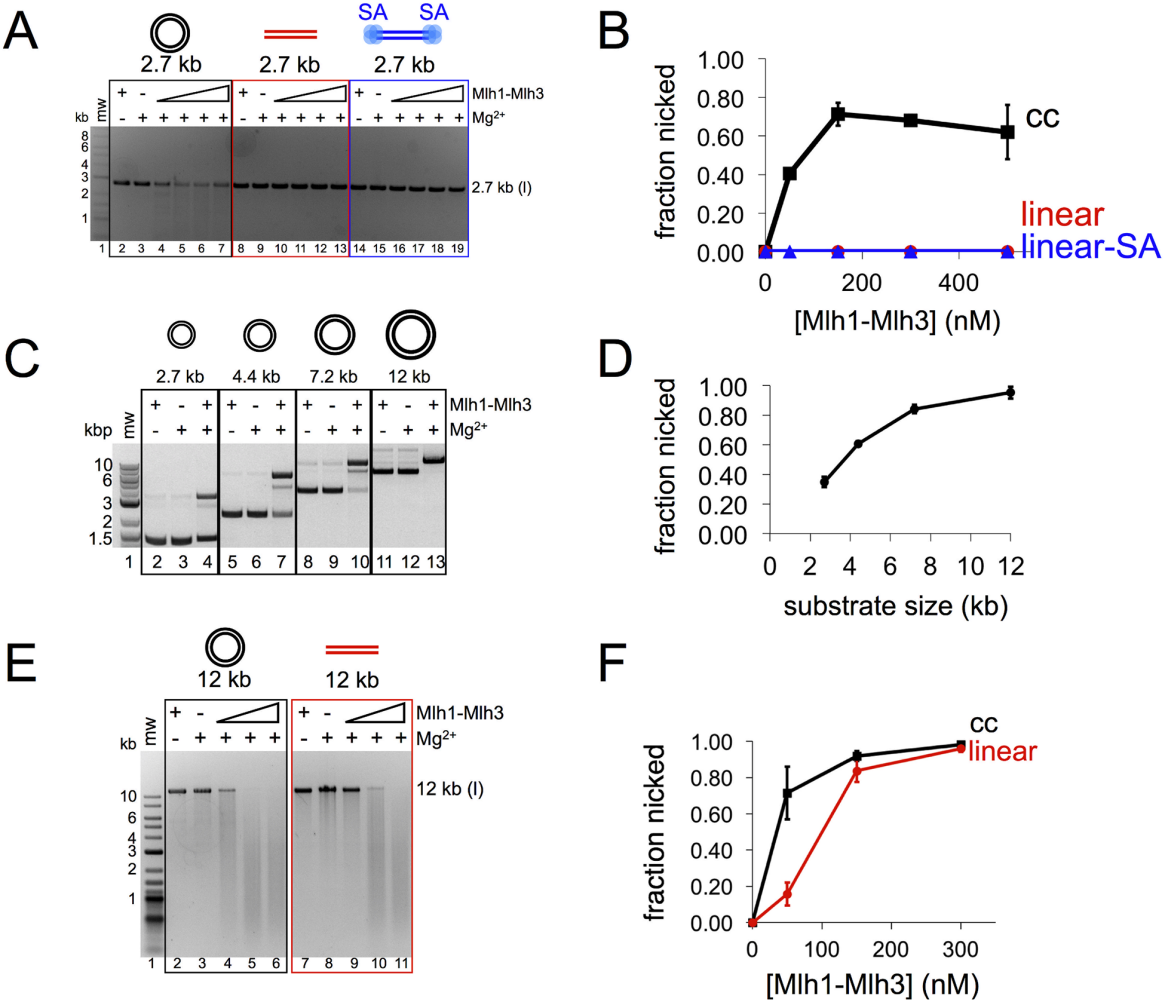
Mlh1-Mlh3’s endonuclease activity requires a continuous substrate and increases as substrate size increases. (A) Denaturing agarose analysis of yeast Mlh1-Mlh3 nicking on circular pUC18 (linearized prior to gel loading) (2.7 kb; black), *Hin*dIII linearized pUC18 (red), and *Hin*dIII linearized pUC18 with streptavidin (SA) bound to ends (blue). Migration of linearized substrate (l) is indicated. (B) Average of two separate experiments: fraction nicked defined as fraction of substrate lost plotted against yeast Mlh1-Mlh3 concentration; error bars represent the standard deviation between three experiments. (C) Top: native agarose gel electrophoresis analysis of yeast Mlh1-Mlh3 (150 nM) endonuclease activity on circular substrate ranging from 2.7 kb to 12 kb. The concentration of nucleotide in each reaction is 15 μM. (D) Quantification of nicking in lanes 4, 7, 10, and 13 in C averaged from three separate experiments. Error bars indicate standard deviation. (E) Denaturing agarose analysis of yeast Mlh1-Mlh3 nicking on 12-kb circular DNA (black) and *Hin*dIII linearized 12 kb substrate (red). (F) Average of three separate experiments; error bars represent standard deviation. All nicking reactions were carried out for 60 min.

We tested if Mlh1-Mlh3 would display a similar endonuclease activity on a 2.7 kb linear duplex identical in size and sequence to a supercoiled circular substrate (Fig 2A and 2B and S2A and S2B Fig). While we observed significant activity on the circular substrate, we were unable to observe nicking on the linear substrate using either Mg^2+^ or Mn^2+^ as a metal cofactor. To determine if the lack of endonuclease activity on the linear substrate was caused by Mlh1-Mlh3 sliding off of the ends of the substrate before it could nick, we biotinylated the 3′ ends of the linearized plasmid and attached streptavidin to block the ends. Attaching streptavidin to the ends of the linear DNA did not restore Mlh1-Mlh3 nicking (Fig 2A and 2B). The preferential nicking of circular double-stranded DNA (dsDNA) was not due to the negatively supercoiled topology, because both relaxed and negatively supercoiled plasmids were nicked with similar efficiencies (S3 Fig).

To better understand Mlh1-Mlh3’s substrate preferences, we tested Mlh1-Mlh3 endonuclease activity on 1.4 to 15 kb circular dsDNA substrates (Fig 2C and 2D, S2C, S2D and S6 Figs; data for 1.4 kb circular substrate are in S5B Fig). Although Mlh1-Mlh3 nicked all circular substrates tested, activity was directly proportional to plasmid size in the presence either Mg^2+^ or Mn^2+^ cofactor. Optimum endonuclease activity for Mlh1-Mlh3 was seen at an ~100:1 ratio of Mlh1-Mlh3 to 2.7 kb plasmid. This finding suggested that multiple Mlh1-Mlh3 molecules interact on the DNA substrate to activate endonuclease activity. It is also consistent with the finding that a large molar excess of Mlh1-Mlh3 is required to nick even the smallest DNA substrate tested (~1,400 bp), which is at least 30-fold larger than the size of DNA required to form a Mlh1-Mlh3-duplex DNA complex in gel-shift assays [27].

**Fig 3:**
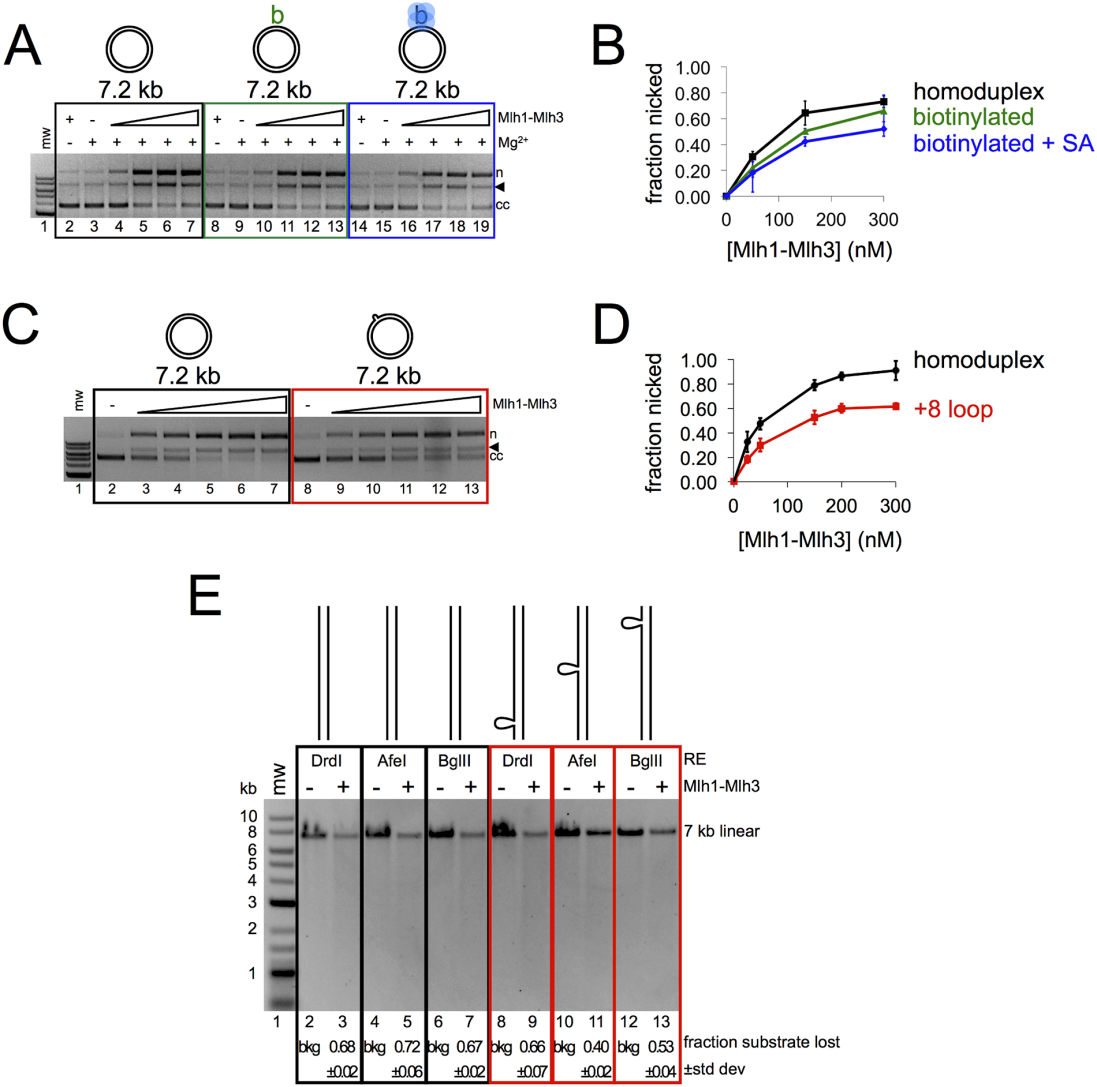
Mlh1-Mlh3’s endonuclease activity is inhibited by a loop mismatch and biotin-streptavidin linkages in plasmid DNA. (A) Mlh1-Mlh3 nicking activity on homoduplex (black), biotinylated (green), or biotin-streptavidin–containing (blue) 7.2 kb circular substrates (15 μM total nucleotide). Lanes 4–7, 10–13, and 16–19 contain 50, 150, 300, and 500 nM Mlh1-Mlh3, respectively. The amount of nicked product (n) and linear product (black triangle) was quantified as a fraction of the total starting closed circular substrate (cc). (B) Average of three separate experiments is plotted. Error bars indicate the standard deviation. (C) Mlh1-Mlh3 nicking activity on homoduplex (black) or +8 loop mismatch–containing (red) 7 kb circular substrate (15 μM total nucleotide). Lanes 3–7 and 9–13 contain 25, 50, 150, 200, and 300 nM Mlh1-Mlh3, respectively. (D) Average of three separate experiments is plotted. Error bars indicate the standard deviation. (E) Mlh1-Mlh3 nicking activity on 7 kb linear substrates containing a +8 loop mismatch 550 (*Drd*I), 3,900 (*Afe*I), or 6,600 (*Bgl*II) base pairs from one end. Average of two experiments is indicated below the gel. See Materials and methods for details. All nicking reactions were carried out for 60 min.

**Fig 4:**
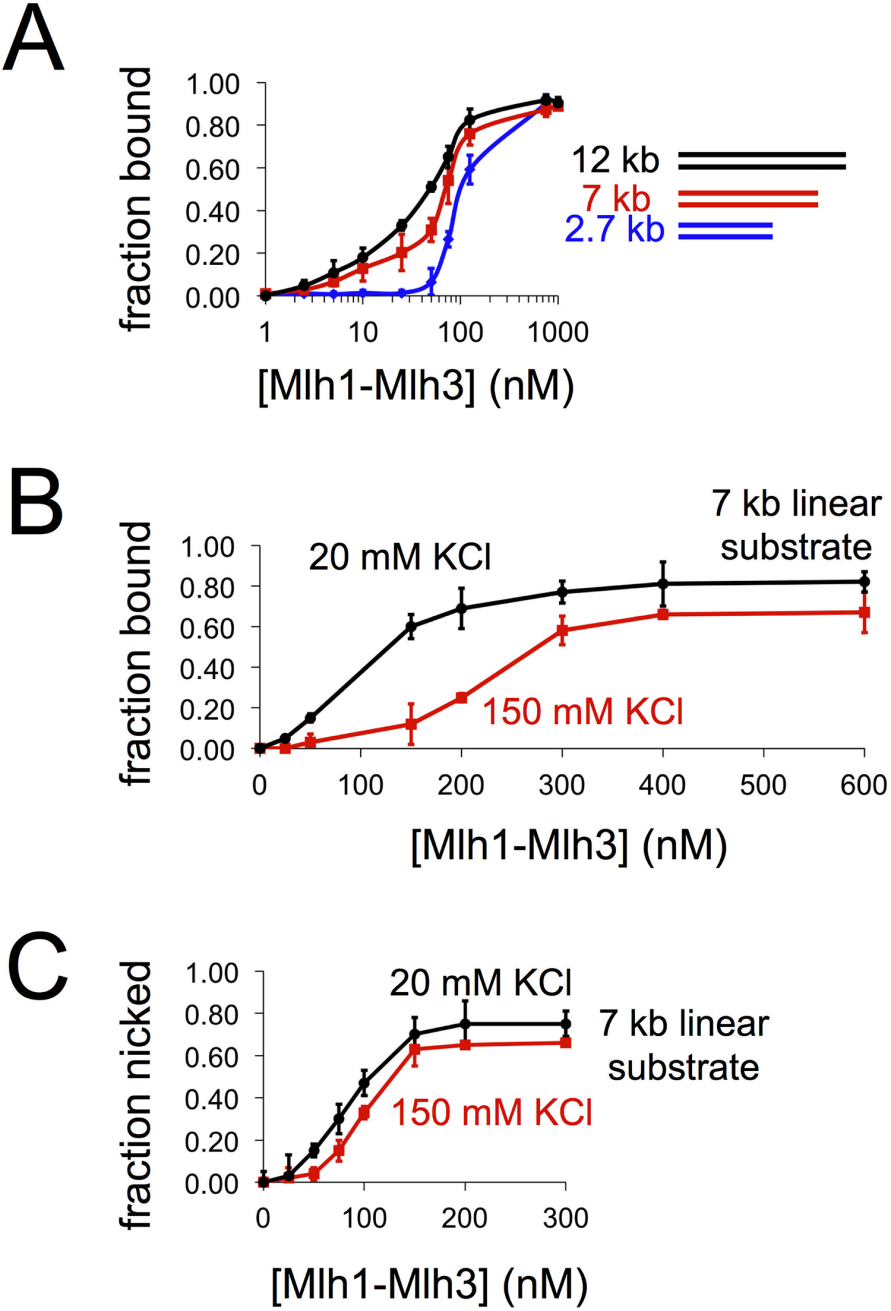
Mlh1-Mlh3 binding to DNA displays a sigmoidal pattern. (A) Nitrocellulose filter binding reactions for linearized 2.7 kb, 7 kb, or 12 kb DNA. The fraction of nitrocellulose-bound DNA was plotted as a function of Mlh1-Mlh3 concentration on a log-scale. For the 2.7-kb substrate, data are for four independent experiments. For the 7 kb and 12 kb substrates, three replicates were performed. All reactions were carried out for 10 min. The average is plotted with error bars representing the standard deviation between experiments. (B) Nitrocellulose filter binding reaction. Increasing amounts of Mlh1-Mlh3 were incubated with 7 kb linear DNA in a buffer containing either 20 mM KCl or 150 mM KCl (all buffers contained 2 mM Mg^2+^). The reaction was performed in triplicate. The average and standard deviation (error bars) are shown here. (C) Nicking reaction. 7 kb linear DNA incubated with increasing amounts yeast Mlh1-Mlh3 in a buffer containing either 20 mM KCl or 150 mM KCl. Endonuclease conditions are identical to those previously published, with a reaction time of 60 min [26]. Activity was assayed for two independent experiments and quantified by alkaline agarose gel electrophoresis.

Interestingly, Mlh1-Mlh3 can cleave both circular and linear DNA substrates that are at least 7 kb in size (Figs 2E, 2F, 3E and 4C). This result correlates with the data presented in Fig 2C and 2D and shows that Mlh1-Mlh3 has higher DNA cleavage activity on larger substrates but less overall activity on linearized DNA. One way to explain this is that on circular DNA, Mlh1-Mlh3 can bind to any initial site and form a polymer. On linear DNA, however, some sites are closer to an end compared to others. If Mlh1-Mlh3 initially nucleates near the end of linear DNA, the polymer length sufficient for robust cleavage may not be achieved. This could explain why Mlh1-Mlh3 can nick a 12 kb linear substrate but not a 2.7 kb linearized substrate, as the majority of random nucleation sites within a 12-kb-long linearized plasmid will be located away from a DNA end. These data and the fact that Mlh1-Mlh3 can nick 1.4 kb and 2.7 kb circular substrates provide clues for the critical length of an Mlh1-Mlh3 polymer needed for nuclease activity (see Discussion).

The above observations suggested that disruptions in DNA that interfere with polymer formation would inhibit Mlh1-Mlh3 endonuclease activity. To test this, we synthesized a 7.2 kb circular substrate containing a single biotin moiety [35]. We titrated Mlh1-Mlh3 into reactions containing either homoduplex 7.2 kb circular DNA or 7.2 kb circular DNA with a biotin with or without bound streptavidin. All substrates were prepared by primer extension in an identical manner. In these experiments, we found that the biotin alone was sufficient to inhibit the reaction by a small amount, but the addition of bound streptavidin inhibited nicking efficiency ~1.5-fold (Fig 3A and 3B). These data suggest that a relatively small perturbation in a large DNA sequence can inhibit Mlh1-Mlh3 activity. As indicated above, we observed a similar result when an eight-nucleotide loop was incorporated into the substrate (Fig 3C and 3D). In addition, we found that the loop insertion inhibited Mlh1-Mlh3 to a greater extent when present at the center of a linear substrate compared to an end, which is consistent with Mlh1-Mlh3 polymer formation being an important requirement for activating its endonuclease activity (Fig 3E).

We next tested whether Msh2-Msh3, which preferentially binds to loop mismatches and stimulates Mlh1-Mlh3 endonuclease activity on duplex DNA [26], would overcome the inhibitory effect that the loop mismatch has on Mlh1-Mlh3 activity (S4C Fig). We also tested whether Mlh1-Mlh3 nicking on the +8 loop circular substrate was localized to the mismatch. This was accomplished by incorporating a radiolabel into the circular substrate ten base pairs away from the mismatch either on the same or opposite strand as the mismatch (S4B Fig). Under conditions when we observed Mlh1-Mlh3 stimulation by Msh2-Msh3, we first measured the total amount of nicking on the substrate in the presence and absence of Msh2-Msh3 (S4C Fig, left). We then excised from a denaturing polyacrylamide gel, a 2.3 kb fragment centrally encompassing the mismatch and the radiolabel (S4C Fig, right). On a homoduplex substrate, Msh2-Msh3 increased the amount of nicked product from 15% to 36%. In reactions containing only Mlh1-Mlh3 and the loop substrate, we observed 10% nicking of the substrate. The addition of Msh2-Msh3 increased this level to 21%. If the inhibition of cleavage of the +8 loop substrate was accompanied by shifting the distribution of nicks to positions near the mismatch, we should observe either the appearance of a specific low-molecular-weight band or a loss in a radioactive signal of the 2.3 kb fragment in the denaturing gel corresponding to the amount of substrate nicked in the agarose gel where we detect nicking on the entire substrate. Instead, we observed no significant loss of signal when the radiolabel was on either strand relative to the mismatch, similar to what was observed on the homoduplex substrate. These results indicate that the nicking observed on the +8 loop substrate was random (see Discussion).

Hall et al. observed using atomic force microscopy that yeast Mlh1-Pms1 forms long protein tracts on DNA and that these tracts associate with two regions of dsDNA in a single plasmid that do not appear to require homology [31]. They suggested that yeast Mlh1-Pms1 has higher binding affinity for larger DNA molecules and additionally shows a preference for circular over equivalent linearized DNA substrate because of the propensity of larger and circular molecules for having two regions of dsDNA in close proximity as a result of greater substrate flexibility in solution. It is possible that this is a common property of MLH proteins that is also utilized by Mlh1-Mlh3 (see Discussion). We observed similar properties for human MLH1-PMS2 (S5 Fig).

To directly observe Mlh1-Mlh3’s DNA-binding properties on large DNA substrates, we linearized and radiolabeled 2.7 to 12 kb plasmid DNAs and performed nitrocellulose filter binding assays in the presence of increasing amounts of Mlh1-Mlh3. In these assays, a single protein bound to DNA is sufficient to retain DNA on the nitrocellulose filter and be scored as bound protein. For this reason, the shape of the resulting binding curve can be used only as a rough measure of a protein’s binding properties. This analysis was performed previously to characterize the DNA-binding properties of Mlh1-Pms1 [31]. They observed sigmoidal binding curves, suggesting that Mlh1-Pms1 binds cooperatively to DNA (see [36]). We show here that Mlh1-Mlh3 displayed a modest preference for binding to larger over smaller DNA substrates with K_d_ values of ~40, 65, and 100 nM for 12, 7, and 2.7 kb linear substrates, respectively (Fig 4A). For the 7 kb substrate, when the amount of potassium chloride (KCl) was increased, the protein’s binding curve was sigmoidal, which suggests the presence of multiple Mlh1-Mlh3 molecules (Fig 4B).

**Fig 5:**
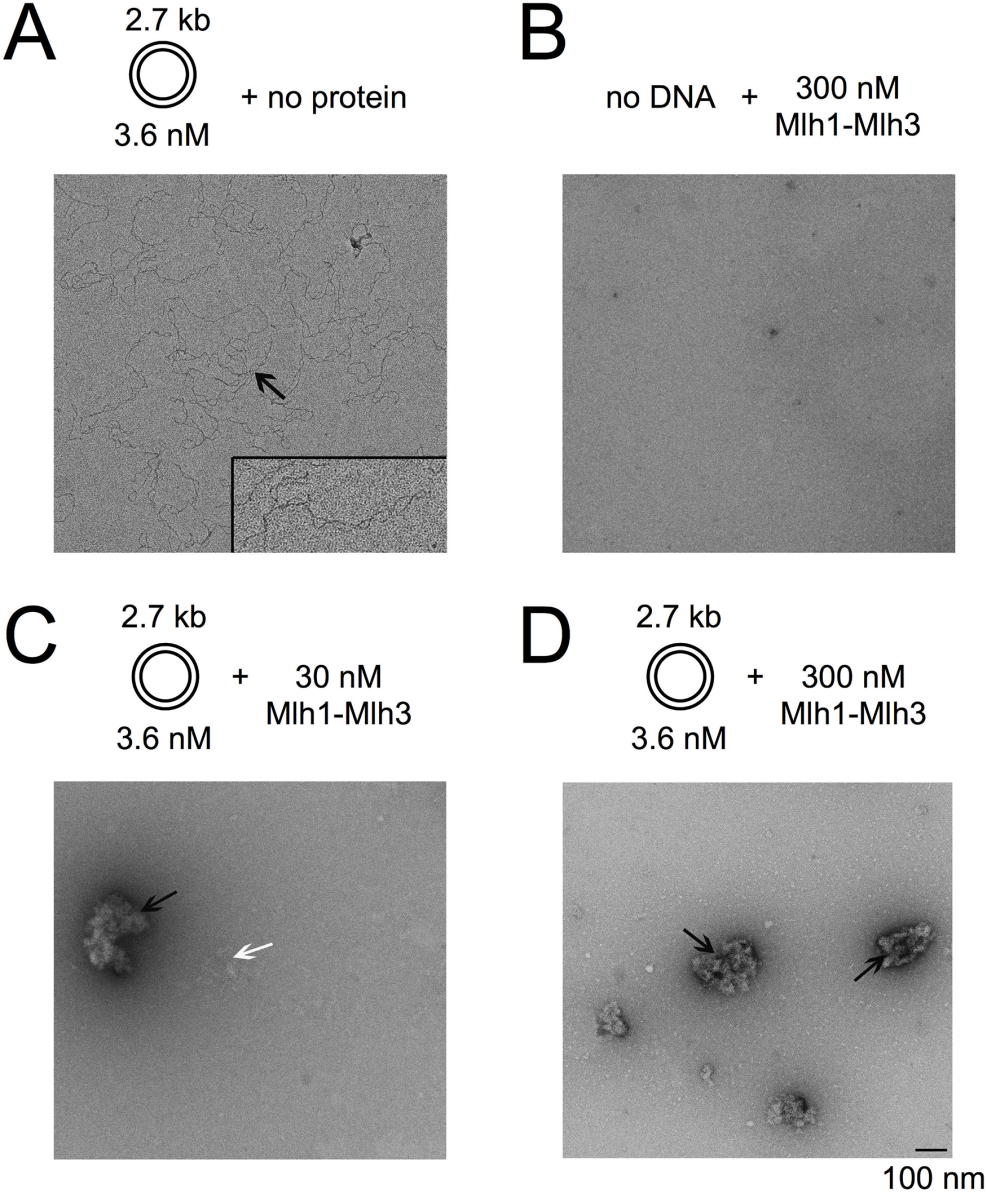
Negative stain images of Mlh1-Mlh3 binding to circular DNA using a K2 direct detector. (A) Representative electron micrograph of sample containing 3.6 nM of 2.7 kb circular DNA in the absence of protein. DNA is indicated by black arrow. A total 50 particles were assessed and all were classified as naked DNA particles. The inset area contains a zoomed view of one of the DNA molecules. The image for this molecule is 2-fold magnified with respect to the entire micrograph in this panel. (B) The electron micrograph shows a sample containing 300 nM Mlh1-Mlh3 in the absence of DNA. (C) Representative electron micrograph of sample containing 30 nM Mlh1-Mlh3 + 3.6 nM 2.7 kb circular DNA. Protein and DNA form both loosely (white arrows) and tightly packed (black arrow) clusters. Naked DNA was also observed in this sample but it is not shown in the electron micrograph. A total of 105 particles were analyzed under these conditions. 50 (48%) were completely condensed clusters, 22 (21%) were partially condensed, and 33 (31%) were naked DNA. (D) Electron micrograph showing sample containing 300 nM Mlh1-Mlh3 + 3.6 nM 2.7 kb circular DNA. Higher concentration of protein induces formation of more condensed protein–DNA clusters (black arrows). Mlh1-Mlh3 protein complexes are also seen in the background of the micrograph. 53 total particles were assessed. Of these, 50 (94%) were completely condensed clusters, 1 (2%) was a partially condensed, and 2 (4%) were naked DNA.

To directly visualize whether multiple Mlh1-Mlh3 molecules bound to DNA are critical for Mlh1-Mlh3 nicking activity, we performed negative staining electron microscopy experiments on samples containing the following mixtures: circular DNA alone (Fig 5A); 300 nM Mlh1-Mlh3 alone (Fig 5B); 30 nM Mlh1-Mlh3 in the presence of DNA, which confers suboptimal nicking activity (Figs 2A, 2B and 5C); and 300 nM Mlh1-Mlh3 in the presence of DNA, which confers robust endonuclease activity on circular DNA (Figs 1A, 2B and 5D). By analyzing multiple grids and multiple locations on these grids, we observed the following: (1) Circular DNA alone displays different degrees of supercoiling but no higher-order structures (Fig 5A), and Mlh1-Mlh3 in the absence of DNA did not show any distinct structures (Fig 5B). (2) At 30 nM Mlh1-Mlh3, small protein–DNA clusters are observed that range from loosely (Fig 5C, white arrow) to tightly condensed (Fig 5C, black arrow). Naked DNA was also observed in this sample but with low frequency. The fact that under limiting Mlh1-Mlh3 concentrations, both partially and tightly condensed protein–DNA clusters were observed supports the intrinsic propensity of Mlh1-Mlh3 to multimerize on DNA. (3) At 300 nM Mlh1-Mlh3, we rarely observed loosely packed clusters or naked DNA. We found that most of the DNA was in tightly packed clusters (Fig 5D). The fact that these conditions resulted in the highest endonuclease activity suggests that the condensed clusters are not inactive coated molecules, but rather active Mlh1-Mlh3 complexes optimally capable to cleave DNA.

Our biochemical analysis suggested that large DNA molecules can accommodate multiple Mlh1-Mlh3 complexes, and interactions among these dimers can activate the endonuclease activity. We hypothesize that polymer formation licenses the Mlh1-Mlh3 cluster to introduce a nick. Our data also suggest that polymer formation can be disrupted by modifications to DNA (Fig 3). Such activities appear distinct from those seen for established HJ resolvases, which recognize and symmetrically cleave branched DNA structures (reviewed in [7]).

**Fig 6:**
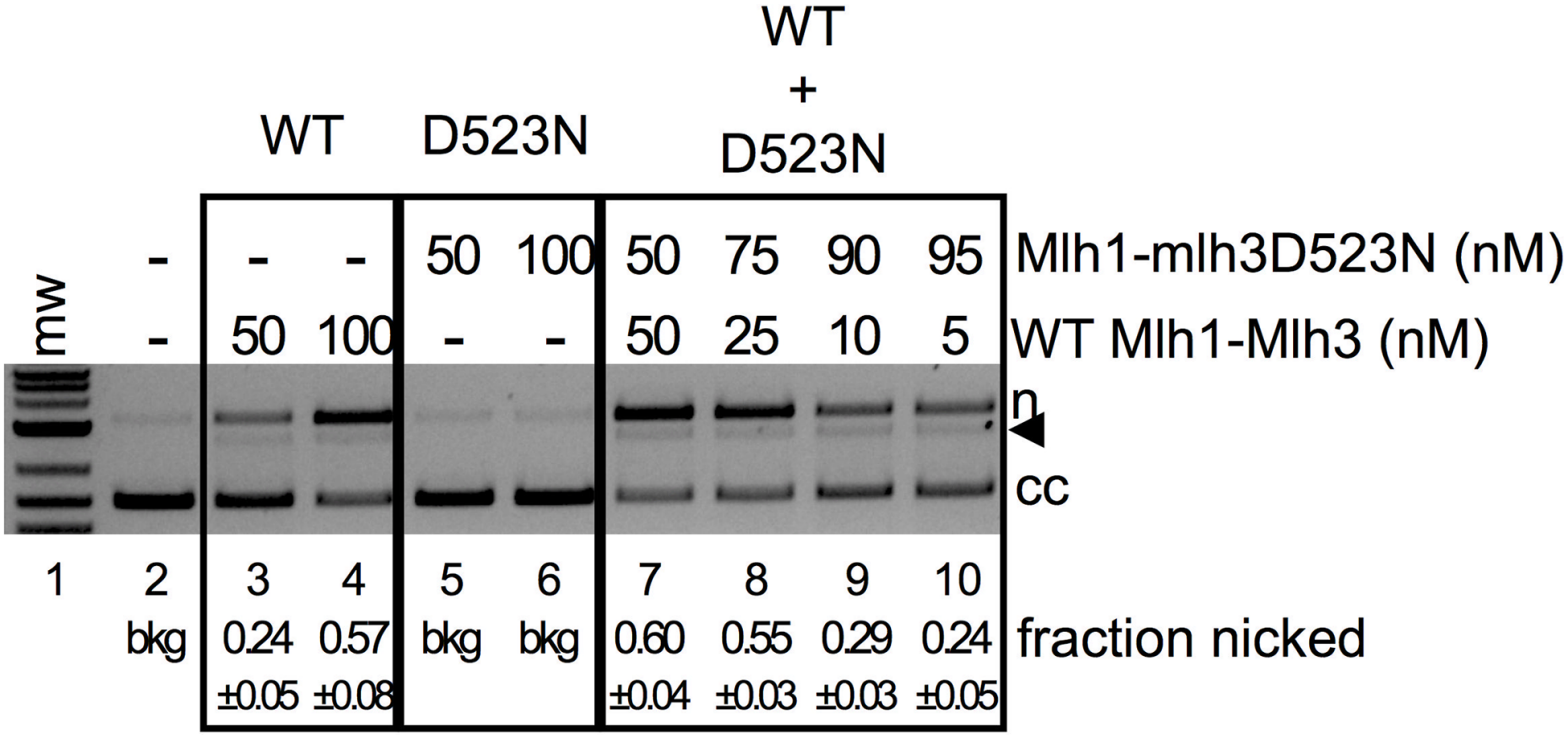
Mlh1-mlh3D523N enhances Mlh1-Mlh3 endonuclease activity. Endonuclease activity was performed with supercoiled pUC18 as described in the Materials and methods. Endonuclease reactions were carried out using the indicated final concentrations of wild-type Mlh1-Mlh3 or Mlh1-mlh3D523N. When both were present, proteins were added to the reaction simultaneously. The average fraction nicked from four experiments is indicated below the gel along with the standard deviation between experiments.

To further test the idea that interactions between Mlh1-Mlh3 dimers activate endonuclease activity, we performed mixing experiments in which the endonuclease dead Mlh1-mlh3D523N complex was added to reactions containing suboptimal concentrations of Mlh1-Mlh3. Mlh1-mlh3D523N, although deficient for endonuclease activity, is an intact heterodimer and retains its DNA binding properties [26,27]. Consistent with this, the *mlh3D523N* allele confers a *mlh3Δ*-like phenotype for meiotic crossing over [19]. These experiments were performed in the linear range for Mlh1-Mlh3 endonuclease activity. As shown in Fig 6, Mlh1-mlh3D523N addition (up to 75% of the MLH complex present) increased Mlh1-Mlh3 endonuclease activity to levels similar to exclusively wild-type complex addition, providing further support for polymer formation being critical for Mlh1-Mlh3 endonuclease function.

### Mlh1-Mlh3 can efficiently make DSBs in a concerted mechanism

The Mus81-Mms4 structure-selective endonuclease is active on HJ substrates containing a nick directly adjacent to a branch point (reviewed in [24]). This discontinuity provides flexibility in the substrate that allows the bound heterodimer to undergo a conformational change to position the branch point in the active site of the complex [37,38]. Mus81-Mms4 then introduces a nick at a discrete point opposite the preexisting nick. Being directed by a preexisting nick in a DNA substrate is a hallmark of Mlh1-Pms1 in partially reconstituted mismatch repair reactions. In MMR, Mlh1-Pms1 introduces a nick on the same DNA strand that contains the preexisting nick, although the position of the nick can be several hundred base pairs away [28,29].

**Fig 7:**
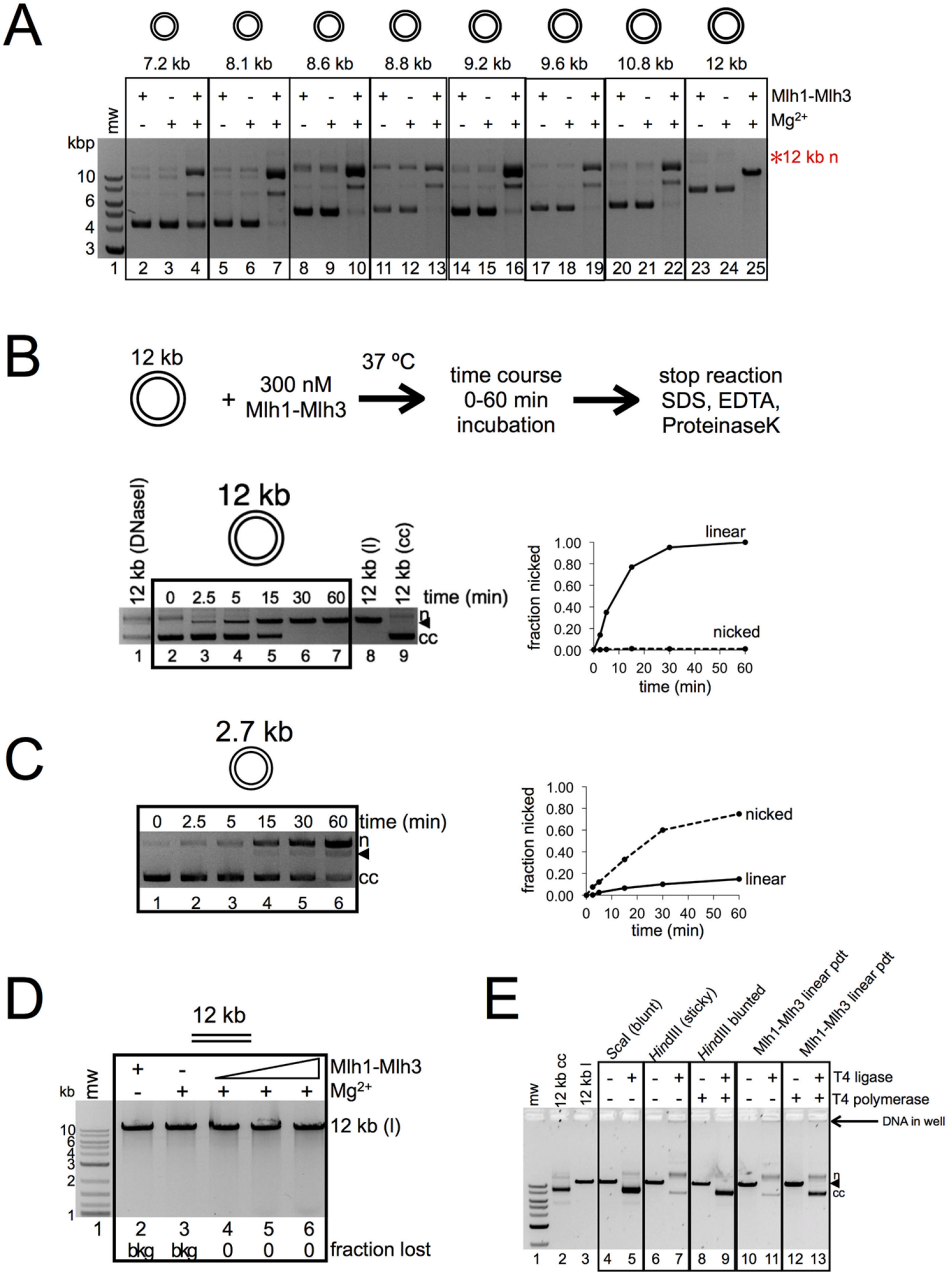
Mlh1-Mlh3 can make DSBs on large DNA substrates. (A) Mlh1-Mlh3 makes exclusively linear product from a closed circular substrate approximately 12 kb in size. Experiments were performed identical to the manner described in Fig 2C using the indicated sized plasmids. Red asterisk indicates location where nicked 12 kb plasmid migrates. (B) 15 μM total nucleotide 12 kb circular substrate was incubated with 300 nM Mlh1-Mlh3 for the indicated period of time. Plasmid linearized with *Hin*dIII was used as a marker for linear product in the lane 8 of the left panel. 12 kb plasmid treated with DNaseI is used as a marker for closed circular, linear, and nicked species in lane 1. Lane 9 is a negative control reaction in which Mlh1-Mlh3 was omitted to indicate migration of closed circular substrate. The plot indicates quantification of the representative gel shown. (C) Experiment is identical to that conducted in B, except 15 μM total nucleotide 2.7 kb circular substrate was used. The plot indicates quantification of the representative gel shown. (D) Native gel analysis of material in Fig 2E lanes 7–11. (E). DSBs made by Mlh1-Mlh3 can be religated. 12 kb linear product from Mlh1-Mlh3 endonuclease assay (“Mlh1-Mlh3 linear pdt”) was gel isolated and incubated with T4 polymerase where indicated with a + (lanes 12–13) followed by T4 DNA ligase where indicated with a + (lanes 11, 13). As controls, 12 kb closed circular plasmid was linearized with either *Sca*I or *Hin*dIII and religated (lanes 4–7) or linearized with *Hin*dIII and blunted with T4 polymerase followed by a religation step (lanes 8–9). Gel-isolated 12 kb closed circular DNA and *Sca*I-linearized DNA were ran in lanes 2–3 as migration markers.

Mlh1-Mlh3 is able to convert closed circular substrate into nicked open circular and linear product (Figs 2C and 3C, for example). The appearance of the linear product suggests that one nick may encourage additional nicking to a site opposite the first nick. We observed that incubating Mlh1-Mlh3 with 12 kb plasmids (pEAE99, pEAO202, pEAE324) resulted in the exclusive formation of linear product with no nicked intermediate, an activity that was also seen for even larger (14 kb-pEAE107, 15 kb-pEAM58) substrates (Figs 2C and 7A, S6 Fig). In time course experiments performed with pEAE99, we did not observe nicked product at any time point assayed (Fig 7B, compare lanes 3–7 to background nicked circle observed in lane 2, DNaseI-treated plasmid in lane 1, and *Hin*dIII-linearized pEAE99 in lane 8). Circular DNA was directly converted into a linear product, suggesting that the two nicks on this large DNA substrate were rapidly made through a concerted mechanism. Consistent with a mechanism that creates concerted nicks to form a double-strand break, the linear fragments created by Mlh1-Mlh3 endonuclease activity could be religated to form closed circular and nicked products, as well as material that failed to enter the well of the gel, much like the religation behavior of linearized product that contains *Hin*dIII overhangs (Fig 7E). When Mlh1-Mlh3 linear products were blunt-ended by T4 DNA polymerase, the amount of closed circular product increased, and the religation appeared to behave more similarly to a blunt-end linear fragment. Curiously, when linearized, pEAE99 was used as a substrate, DSBs were not detected in native agarose gels (Fig 7D), but single-strand nicks were detected in denaturing gels (Fig 2E). We also observed concerted nicks on the 2.7 kb circular substrate; linear product was seen at time points at which nicked product was still being produced, and this was seen in both relaxed and supercoiled plasmid (Fig 7C, lanes 4–6, for example; S3 Fig). Together, these data suggest that polymer formation and possibly close-range DNA interactions are critical for Mlh1-Mlh3 to make DSBs through a concerted nicking mechanism (see below).

Human MLH1-PMS2 also converted a 12 kb circular substrate to linear product but did not do so on smaller plasmid substrates (S5A and S5G Fig). To better understand this observation, we tested MLH1-PMS2 activity on a 2.7 kb closed circular substrate and a substrate prenicked using a commercially available restriction endonuclease [26]. We did not observe the appearance of a linear product using either substrate, despite robust nicking of the closed circular substrate (S5G Fig). Nevertheless, because human MLH1-PMS2 can generate linear product on very large circular substrate, these data suggest that the ability to form a DSB is a general property of the MLH complexes and that Mlh1-Mlh3 does this efficiently.

**Fig 8:**
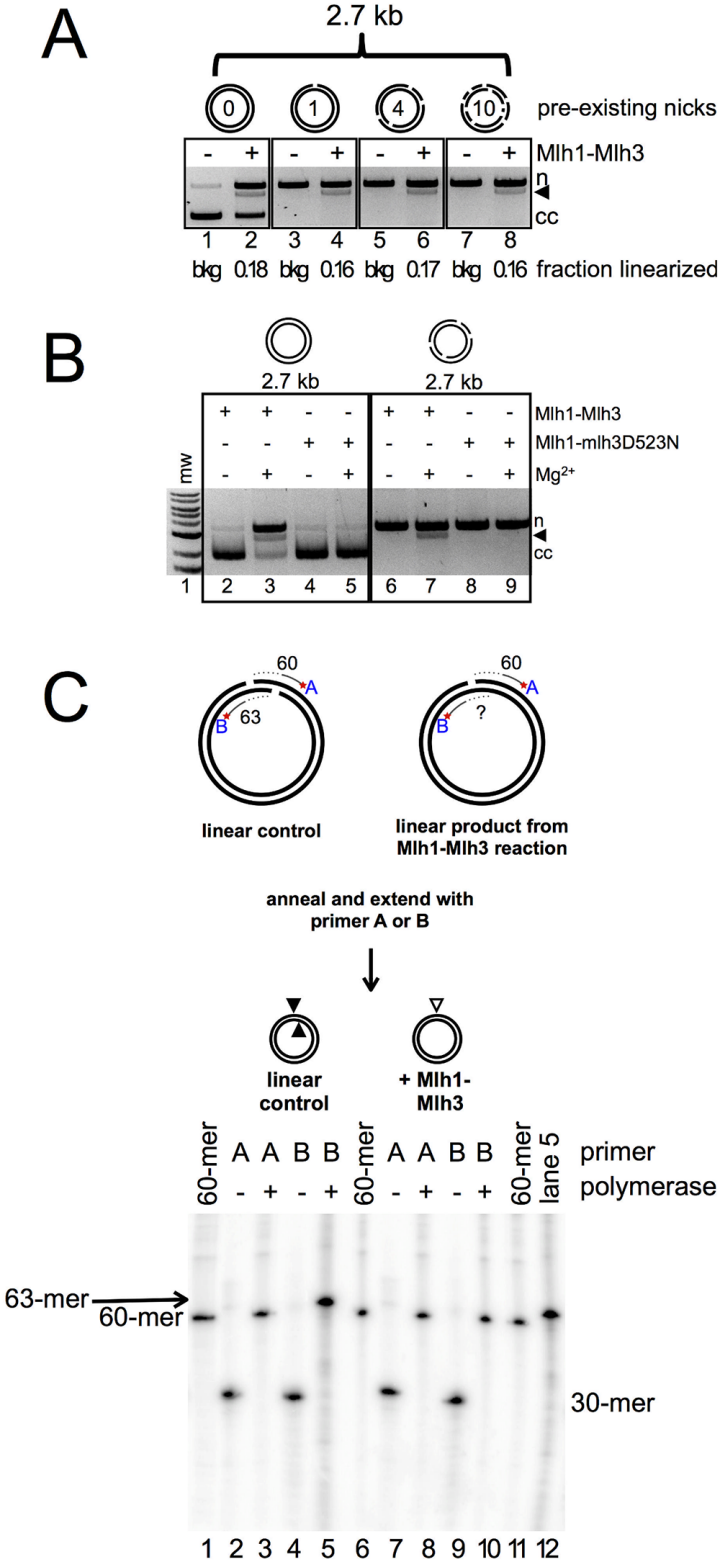
Preexisting nicks act as preferential nicking (but not loading) sites for Mlh1-Mlh3. (A) Mlh1-Mlh3 creates approximately the same amount of linear product regardless of how many preexisting nicks are in the circular substrate. 300 nM yeast Mlh1-Mlh3 on either closed circular 2.7 kb plasmid substrate (0 preexisting nicks), *Nt*.*Bsp*QI-treated substrate (one preexisting nick), *Nt*.*Bst*NBI-treated substrate (four preexisting nicks), or *Nt*.*Alw*I-treated substrate (ten preexisting nicks). Nicked (n) and linear (black triangle) products are shown. (B) Wild-type Mlh1-Mlh3 (300 nM) creates linear product on either closed circular 2.7 kb plasmid substrate or *Nt*.*Bst*NBI-treated substrate, while Mlh1-mlh3D523N (300 nM) is inactive on both. (C) Mapping the formation of Mlh1-Mlh3–induced nick opposite a preexisting nick. Top: experimental setup. Plasmid was either linearized with *Sap*I, which creates a 3-bp overhang, or substrate with a preexisting nick generated by *Nt*.*Bsp*QI was linearized by Mlh1-Mlh3. For each, linear product was gel extracted and annealed to a primer either complementary to the strand with the preexisting nick (primer A) or to the opposite strand (primer B). Primers were extended by T4 polymerase where indicated. Primer extension products were resolved by denaturing PAGE. For the *Sap*I linear control, extension of primer A gives a 60-mer and extension of primer B gives a 63-mer product. Bottom: lanes 1, 6, and 11 are radiolabeled 60-mer used as a primer extension marker. Lanes 2–5 show primer extension for the linear control, while lanes 7–10 show primer extension for Mlh1-Mlh3 linear product. Lane 12 is a duplicate of the material in lane 5.

Previously, we observed Mlh1-Mlh3–dependent conversion of a prenicked substrate to linear DNA using a substrate containing four preexisting nicks [26]. In our reactions, all DNA can be accounted for as either uncut circular, linear, or nicked circular product, indicating that Mlh1-Mlh3 does not introduce an abundance of nicks into the substrate. This suggests that the linear product is not formed upon frequent nicking at random positions. Supporting this, we observed the conversion of nicked into linear DNA under conditions in which only ~50% of closed circular DNA was nicked (Fig 8A lane 2, and in [26]). Once formed, linear product of this size does not support endonuclease activity (Fig 2A). The endonuclease-deficient Mlh1-mlh3D523N was unable to make DSBs, indicating that this is an intrinsic property of the Mlh1-Mlh3 endonuclease (Fig 8B). These observations indicate that the nicks did not occur at a large number of sites on the substrate that would produce linear DNA when two nicks are located close to each other by chance. Rather, our data show that the endonuclease cleavage sites on nicked substrates were not distributed randomly.

We entertained three explanations for the above observations: (1) Preexisting nicks are preferential loading sites for Mlh1-Mlh3 polymer formation and direct Mlh1-Mlh3 to nick directly opposite the preexisting nick. This proposed mechanism is similar to that seen for the Mus81-Mms4 resolvase (reviewed in [24]). (2) Preexisting nicks have no impact on Mlh1-Mlh3’s ability to form a DSB, but the complex introduces two nicks, making a DSB at a site independent of the position(s) of preexisting nicks. This idea is suggested by our finding that linear product is formed to similar extents by using closed plasmid and prenicked plasmid as substrates [26] and by the time course experiments presented in Fig 7B and 7C. (3) In the absence of a preexisting nick, an Mlh1-Mlh3 polymer can introduce a nick that is then used by the active polymer to introduce an additional nick on the opposing strand. In this model, a preexisting nick serves as a preferential site for an Mlh1-Mlh3–generated nick on the opposite strand but does not serve as a preferential loading site. Such a model argues that Mlh1-Mlh3’s endonuclease acts distinctly from Mus81-Mms4 and is supported by the finding that the amount of linear product is similar in reactions containing closed circular or prenicked substrates [26].

To differentiate between the above mechanisms and address whether preexisting nicks are used as preferential substrates for Mlh1-Mlh3, we generated nicked substrates containing one, four, or ten preexisting nicks in the pUC18 plasmid. If preexisting nicks act as preferential loading sites for Mlh1-Mlh3, we would expect an increase in linear product as the number of preexisting nicks increases, and if each nick is used as a recognition site, we would expect to see smaller linear fragments or perhaps a smear on the agarose gel. The amount of linear product was comparable for closed circular substrate or circular substrate with any of the number of preexisting nicks tested (Fig 8A). These data imply that formation of the DSB is likely independent of discontinuities in the DNA substrate and that nicks are not preferential loading sites for an Mlh1-Mlh3 polymer (favoring models 2 and 3).

If model 3 is correct, then we should be able to map a DSB to a specific location in the plasmid. On closed circular DNA, Mlh1-Mlh3 generated nicks are not made at sequence-specific locations or near any known secondary structure on the plasmid. To determine where Mlh1-Mlh3–generated nicks are located relative to a preexisting nick, we used a substrate that contains one nick introduced by a restriction-nicking endonuclease. We incubated this substrate with Mlh1-Mlh3, isolated the linear product, and then annealed primers either complementary to the strand with the restriction nick (primer A) or complementary to the opposing strand (primer B) approximately 60 nt away from the restriction nicking site. A primer extension assay was then performed. In this assay, primer extension terminates at the site of a nick, allowing us to determine if Mlh1-Mlh3 is nicking immediately opposite the preexisting nick (Fig 8C, top). Because of the low amount of linear product generated from this substrate (Fig 8A), we surmised that the preexisting nick does not preferentially act as a loading site for Mlh1-Mlh3. If, however, Mlh1-Mlh3 loaded near the preexisting nick by chance uses a single preexisting nick to direct a nick to a specific site in the opposite strand, we expected to detect a discrete band that would be visible when generated in the primer extension assay and analyzed by denaturing polyacrylamide gel electrophoresis (PAGE). If the preexisting nick only directs nicking to the region but not to a specific nucleotide, we would observe a smear on the gel. If Mlh1-Mlh3 does not use the preexisting nick as a preferred site and the DSB occurs completely independently of the preexisting nick, we will observe extension of the primer to a site much farther than 60 bp away from the preexisting nick on the 2.7 kb substrate.

As a control, we performed the primer extension assay with substrate generated by using a restriction enzyme that linearizes the plasmid by cutting at the site of the preexisting nick and at a site 3 bp away (Fig 8C, top). For the linear control, when primer A was extended, a 60 nt product formed (Fig 8C, bottom, lane 3). When primer B was extended, a 63-nt nucleotide product formed (Fig 8C, bottom, lane 5). For the linear product generated by the Mlh1-Mlh3 reaction, when primer A was extended, a 60-nt product formed (Fig 8C, bottom, lane 8). This corresponds to the site of the preexisting nick. When we extended primer B, we observed a single band at approximately the same intensity as the unextended primer, migrating at ~60 nt (Fig 8C, bottom, compare lane 10 to 9 for intensity and lane 10 to 11 and 12 for size).

The above data indicate that if the Mlh1-Mlh3 complex is loaded near a preexisting nick, it uses the nick to direct endonuclease activity to a specific site precisely opposite this initial precursor nick when present in the substrate. Again, because of the low yield of this linear product, we do not believe that this is a preferred loading site for the polymer. Together, these data suggest that an active Mlh1-Mlh3 polymer can introduce a DSB made by concerted nicks in a mechanism distinct from Mus81-Mms4. If loaded by chance near a preexisting nick, however, the nick can be used as a landmark for Mlh1-Mlh3 to introduce a nick on the opposite strand. With no preexisting nick, nicking is otherwise random, as observed by analysis with denaturing gels (Figs 2A, 2E and 3E). If there were hotspots for nicking on covalently closed substrate, discrete bands would be observed in these gels as opposed to a smear. These observations also provide a hint for how Mlh1-Mlh3 could be directed by a loading factor to specifically cleave recombination intermediates such as dHJs (see Discussion).

### Mlh1-Mlh3 is capable of cleaving DNA that is captured in an active complex (acting in trans)

**Fig 9:**
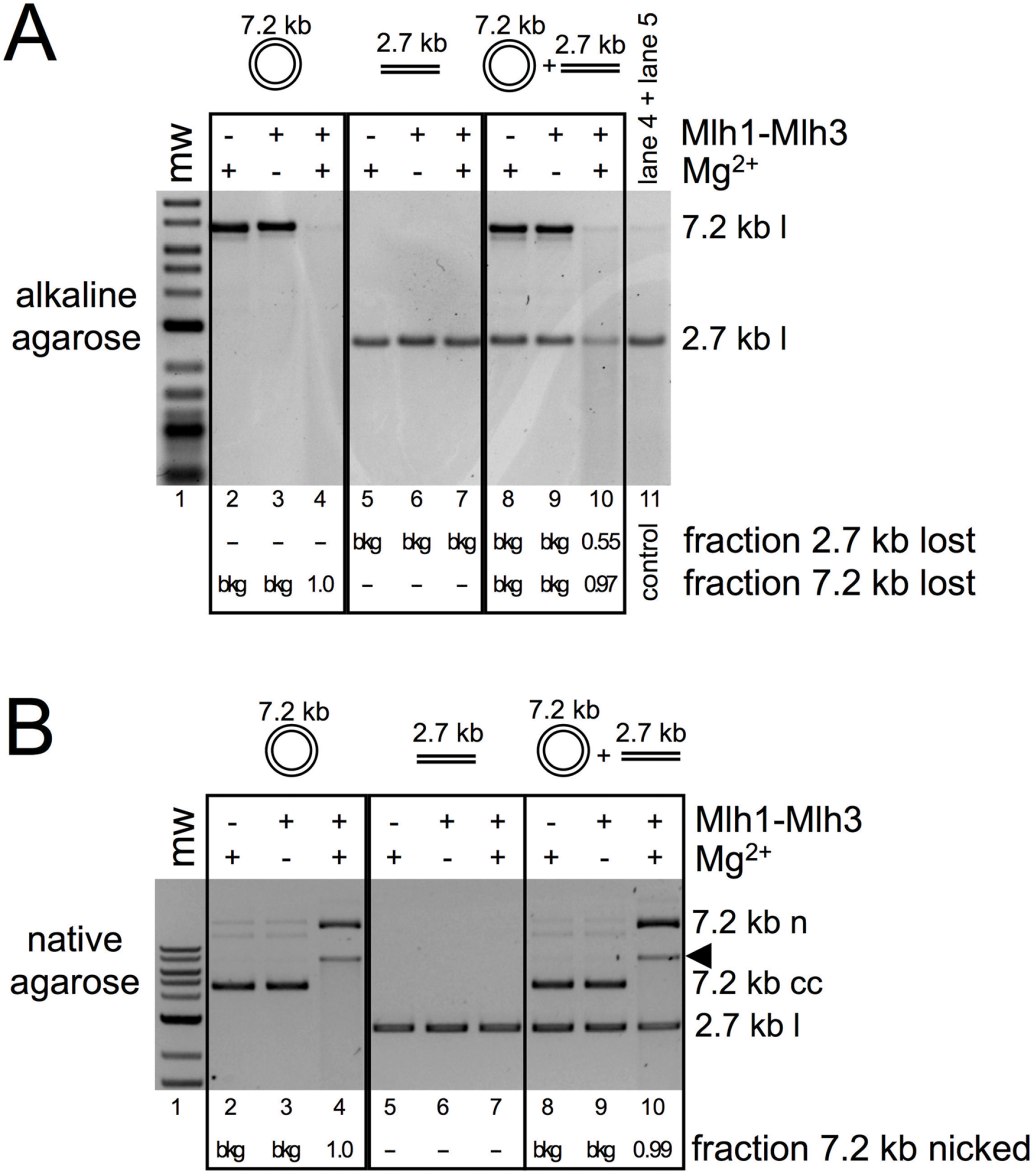
An activated Mlh1-Mlh3-DNA complex can nick DNA in trans. 0.7 nM 7.2 kb closed circular M13mp18 phagemid and 1.8 nM 2.7 kb linear pUC18 substrate were incubated with 300 nM Mlh1-Mlh3 under standard endonuclease assay conditions either in isolation or within the same reaction as indicated. (A) Reaction products were run on an alkaline agarose gel. The 7.2 kb substrate was linearized with *Hin*dIII prior to loading in the alkaline agarose gel. The material in lane 4 and 5 was mixed and run as a control in lane 11 to demonstrate the readout for a negative result. The fraction of DNA nicked was measured by determining the band density in either the 7.2 kb linear band or the 2.7 kb linear band by subtracting the density in a region immediately above the band as background and comparing it to the band densities in the negative controls. (B) Prior to linearization with *Hin*dIII, 10 μL of each reaction was removed and run on a native agarose gel.

The finding that Mlh1-Mlh3 can create concerted DSBs on large circular but not equivalent linear substrates where it made nicks, and that it did not make nicks on smaller linear substrates, suggested that close-range interactions and/or synapsis between DNA molecules could license Mlh1-Mlh3 polymer to display endonuclease activity “in trans.” To test this idea, we performed reactions in which a closed circular 7.2 kb substrate, which is nicked by Mlh1-Mlh3, and a 2.7 kb linear substrate, which is not nicked, were incubated together with Mlh1-Mlh3. As shown in Fig 9, the 2.7 kb linear substrate was nicked only when incubated in the presence of the 7.2 kb closed circular substrate. This observation is consistent with an Mlh1-Mlh3-DNA complex being able to interact with DNA substrates in trans (see Discussion).

## Discussion

Little is known about the mechanism by which Mlh1-Mlh3 acts to resolve meiotic recombination intermediates to form COs. This has been a challenging effort because Mlh1-Mlh3 has little in common with the well-characterized structure-selective endonucleases (e.g., Mus81-Mms4, Slx1-Slx4, and Yen1), both in terms of homology and intrinsic behavior in vitro. The absence of a biochemical paradigm provided by other structure-selective endonucleases makes it difficult to model Mlh1-Mlh3’s role in HJ resolution. Mlh1-Mlh3 likely relies on other protein factors, including Msh4-Msh5, to recruit and coordinate endonuclease activity and collaborates with other factors to spatially and temporarily coordinate the resolution of dHJs into COs in a mechanism that is distinct from the previously established archetypes set by structure-selective endonucleases.

**Fig 10:**
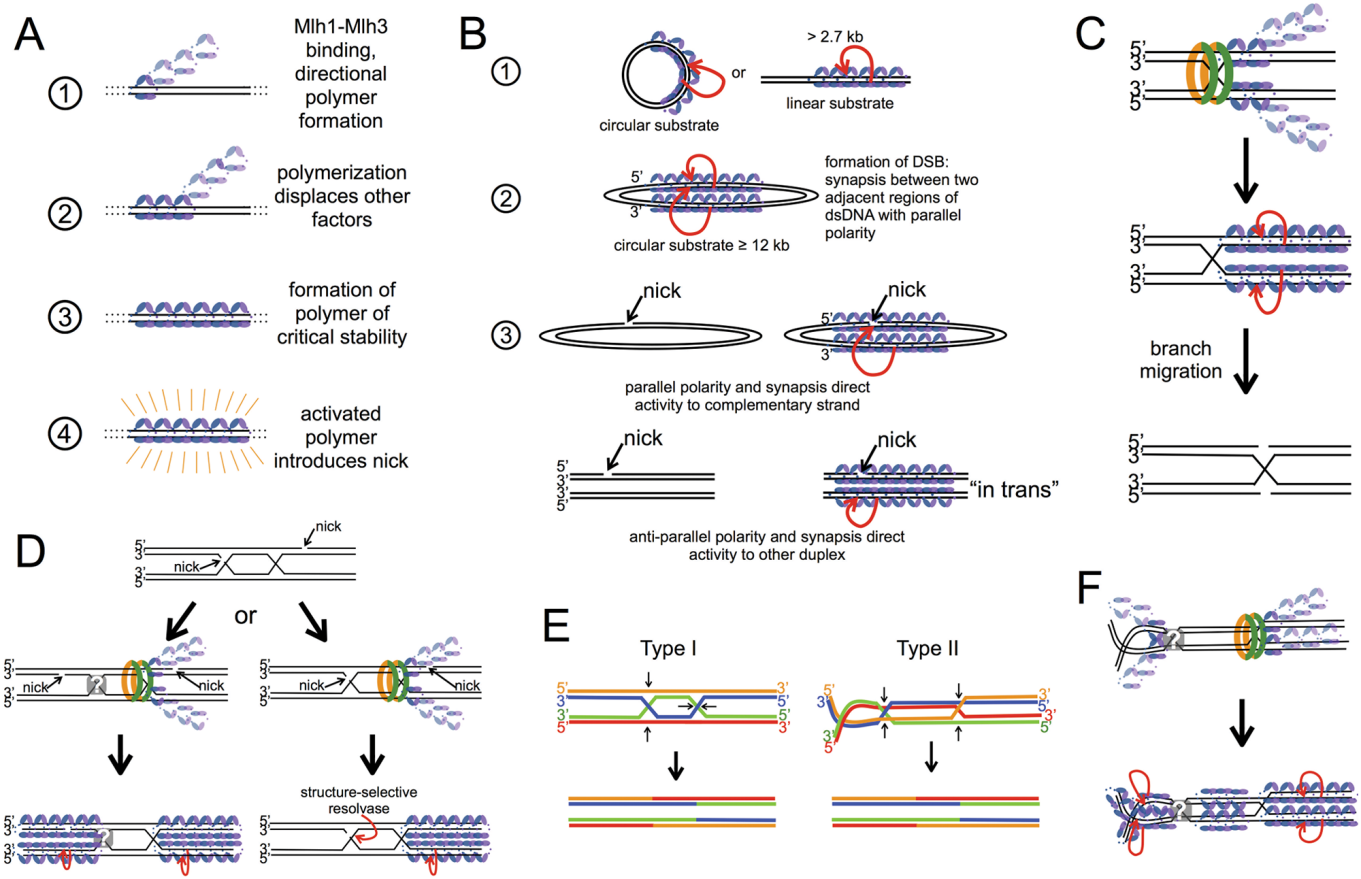
Model for Mlh1-Mlh3 activation. (A) Model for Mlh1-Mlh3’s (blue and purple heterodimer) endonuclease activation dependent on polymerization of the protein. (1) Mlh1-Mlh3 binds DNA and forms a polymer with a specified polarity. (2) Polymer formation proceeds and if other factors are present, they are displaced. (3) A polymer is formed of the critical length and stability for activation. (4) The activated polymer can introduce a nick into one strand of the duplex DNA. See Results and Discussion for details. (B) Mlh1-Mlh3 activation in the context of in vitro substrates. Red arrow indicates nicking activity by an active polymer. (1) An Mlh1-Mlh3 polymer can form on circular or linear DNA. For linear DNA, the molecule must be a length critical for polymer stability. (2) On circular substrate that is at least 12 kb, Mlh1-Mlh3 binds to adjacent regions of duplex DNA, leading to DSB formation. (3) Top: on circular substrates with preexisting nicks, polymers form and interactions between polymers direct Mlh1-Mlh3 to nick opposite the preexisting nick. Bottom: nicking in trans. If the adjacent regions of dsDNA do not have the same polarity, interactions between polymers may occur with a different orientation, promoting nicking to the duplex that does not contain a preexisting nick. (C) Hypothesis for how in vitro observations can be applied to understand HJ resolution. Bound Msh4-Msh5 recruits Mlh1-Mlh3 to form a polymer. Polymer formation and relative polarities of the duplex arms activate endonuclease activity to cleave strands with the same polarity. Branch migration could position nicks directly at the junction. (D) Hypothesis for how Mlh1-Mlh3 may resolve dHJs in vivo if the dHJ is unligated. The mode of resolution is different depending on where synthesis terminates. The grey square with a white question mark indicates unknown occupancy of one of the junctions. Mlh1-Mlh3 is recruited by Msh4-Msh5, which is then displaced by the Mlh1-Mlh3 polymer. The relative polarities of the two duplexes and the nicks resulting from DNA synthesis direct asymmetric resolution with respect to the two junctions. Cleavage could take place near the junction or some distance away. See Discussion for additional details. (E) Two possible conformations of a dHJ. Small black arrows in the top of the panel indicate where nicks would need to form to produce the CO products in the bottom of the panel. See Discussion. (F) Hypothesis for how Mlh1-Mlh3 may resolve type II dHJs in E. Mlh1-Mlh3 is recruited by Msh4-Msh5, which could be displaced by Mlh1-Mlh3. Polymer formation activates the endonuclease to cleave. Cleavage in this model can occur symmetrically with respect to both junctions, either near the junction or at a site away from the branch point.

Here, we showed that Mlh1-Mlh3 polymer formation is a requirement for its in vitro endonuclease activity (Fig 10A and 10B), and that Mlh1-Mlh3 is capable of making DSBs through a concerted nicking mechanism (Fig 10B). Strikingly, we observed that the Mlh1-Mlh3 polymer cleaves long duplex DNA but not small DNA substrates containing loop and branched structures. These observations are reminiscent of the involvement of multiple Mlh1-Pms1 molecules during MMR [30] and the finding that Mlh1-Pms1 does not nick mismatched DNA directly at the site of the mismatch [28,29]. For this reason, we cannot exclude the possibility that such activities may be more critical for mismatch correction than crossing over. The presence of distinct Mlh1 and Mlh3 foci at CO sites in vivo also supports a necessity for multiple Mlh1-Mlh3 heterodimers being required for resolution, since a large number of molecules are needed for foci to be visible. The requirement for polymerization for nuclease activation provides a regulatory mechanism to precisely control nuclease activity. Such a model also predicts that the CO endpoints that involve Mlh1-Mlh3 may be different than those seen by resolution with structure-specific endonucleases that make precise incisions, as DNA cleavage may occur at locations away from the junctions analogous to MMR mechanisms. DNA cleavage followed by branch migration is sufficient to result in CO products (Fig 10C).

Taken together, our data argue strongly against Mlh1-Mlh3 acting on HJs using a Mus81-Mms4–like mechanism. Mus81-Mms4 and other structure-selective endonucleases recognize branched molecules and cleave at precise, discrete locations at the branch points to resolve them. Based on molar ratios and substrate requirements in vitro, there are no polymerization requirements for these canonical resolvases (reviewed in [24]). Mus81-Mms4 and Yen1’s endonuclease activities are also sensitive to their phosphorylation state [25]. Our Mlh1-Mlh3 expressed in *Sf*9 cells was unaffected by phosphatase treatment, which may suggest that Mlh1-Mlh3 is not regulated by phosphorylation, although we cannot rule out an effect of phosphorylation by cognate factors during meiosis in vivo or other posttranslational modifications such as SUMOylation. It should be noted that protein components involved in formation of the synaptonemal complex and stabilization of Msh4-Msh5 have been found to be SUMOylation enzymes (reviewed in [39]) [40–44]. It is possible that such a modification could have an as yet unknown regulatory function on Mlh1-Mlh3, though posttranslational modifications have yet to be implicated in the activity of other MLH family complexes.

Our data indicate that Mlh1-Mlh3 is unlikely to act directly on Holliday junctions and mismatched substrates. We showed that incorporating a +8 loop mismatch into a plasmid substrate has an inhibitory effect on endonuclease activity and does not localize it, and cruciforms present in plasmids are not substrates for Mlh1-Mlh3 cleavage [27]. These observations suggest that HJs and mismatched substrates are not likely to be the preferred in vivo substrates for the Mlh1-Mlh3 endonuclease. Previously, we showed that Mlh1-Mlh3 has a binding preference for oligonucleotide substrates containing a +8 loop mismatch and a HJ over homoduplex DNA but that the binding affinity differences in buffers used in our endonuclease assay were modest [26,27]. However, in the oligonucleotide competition endonuclease assay presented here (Fig 1), HJ and +8 loop–containing oligonucleotide substrates acted as very strong competitors relative to homoduplex DNA, which was an ineffective competing substrate compared to plasmid DNA. Although having preferences for certain structures, Mlh1-Mlh3 is an overall efficient DNA-binding complex [26,27]. Mismatch recognition factor Msh2-Msh3, while preferring 8-nt loop mismatches, does not bind mismatch DNA particularly tightly (K_d_ ~50 nM for loop mismatch; K_d_ ~200 nM for homoduplex) [45]. In experiments where an 8-nt loop mismatch was incorporated into a 7.2 kb plasmid substrate, it should be noted that the portions of the plasmid substrate that do not contain the mismatch may serve as competitor DNA (S4 Fig). Since Mlh1-Mlh3 binds DNA at least as tightly as Msh2-Msh3, it follows that Mlh1-Mlh3 may be competing with Msh2-Msh3 for the loop mismatch as well as the remaining duplex DNA, which is a possible explanation for why we do not observe the ability of Msh2-Msh3 to direct Mlh1-Mlh3 nicking to sites near the mismatch.

### A model to explain how Mlh1-Mlh3 acts in vitro

We hypothesize that Mlh1-Mlh3 forms a polymer on DNA to activate its endonuclease activity (Fig 10A). We suggest that there is a critical polymer length (on the order of 1 kb; S5B Fig) needed to activate the endonuclease activity that is achieved more readily on a circular substrate where all initiating sites are equivalent. It is important to note that one nick is sufficient to convert closed circular substrate to nicked product, and because nicking occurs in random locations on the plasmid, quantifying the number and location of nicks is not feasible. This is evidenced by the lack of a discrete product in denaturing agarose gels (Figs 2A, 2E and 3E). It should also be noted that our experiments suggest Mlh1-Mlh3 makes only a few nicks per substrate molecule. The entirety of the DNA is accounted for after the reaction as either uncut closed circular substrate, nicked circular product, or linear product. If an abundance of nicks were generated, we would observe loss of DNA density in the native agarose gels. Analysis by alkaline agarose gel suggests that nicks are being made in both strands of the duplex because we observe loss of DNA density in these experiments when high concentrations of Mlh1-Mlh3 are added to the reaction.

Our observation that a 2.7 kb circular plasmid can be nicked but the linearized form cannot (Fig 2A and 2B) indicates that the Mlh1-Mlh3 polymer is directional (Fig 10B). Without directionality, one would expect a polymer to form on the linear substrate in different orientations and thus be able to nick the substrate. Although we do observe binding to this linearized substrate, it is insufficient to promote nuclease activity. Together, these data suggest that a unidirectional polymer is required for endonuclease activation.

Why does Mlh1-Mlh3 make DSBs on large circular plasmids but nick smaller ones? Hall et al. showed through atomic force microscopy that yeast Mlh1-Pms1 forms polymers on plasmid DNA and can simultaneously interact with two regions of the substrate [31]. One way to explain this observation is that a single MLH heterodimer simultaneously interacts with adjacent regions of dsDNA. Alternatively, polymers of MLH proteins located at two different locations on a plasmid DNA interact with each other (Fig 10B, panel 2). An observation similar to that made for Mlh1-Pms1 has been made for type IB DNA topoisomerases (TopIBs) [46,47]. Using both atomic force microscopy and electron microscopy, TopIB, in the presence high protein concentrations relative to DNA, forms a polymer on DNA and brings distant, noncontiguous regions within the same circular DNA molecule into close proximity (intramolecular synapsis). When a linear substrate was used, regions of DNA not necessarily within the same molecule were synapsed (intermolecular synapsis). Analysis of a cocrystal structure of TopIB in complex with DNA showed that TopIB has two distinct DNA-binding regions and that a single TopIB polymer can simultaneously interact with two regions of dsDNA [48], in contrast to a model in which two polymers, each interacting with DNA, form a complex. It will be valuable to perform analogous experiments with the MLH proteins to determine if they undergo an intramolecular synapsis mechanism similar to that seen for TopIB.

We observed a substantial overlap in biochemical properties of Mlh1-Mlh3 and MutLα, both of which act in MMR [32]. Similar to Mlh1-Pms1, Mlh1-Mlh3 may associate with two nearby regions of dsDNA, possibly through a synapsis-type mechanism in which two nucleoprotein complexes interact (Fig 10B, panel 2). Larger substrates are more likely to accommodate multiple Mlh1-Mlh3 polymers of sufficient length to activate nicking. These regions of DNA coated with Mlh1-Mlh3 may then be able to interact with one another on these large substrates. Such properties could thus account for the exclusive conversion of closed circular substrates into linear products for plasmids greater than 12 kb, perhaps by creating a nucleoprotein conformation that restricts nicking activity to one of the two interacting duplexes. Our observation that a small linear substrate can be nicked only when incubated in the presence of a larger closed circular substrate is consistent with the idea that Mlh1-Mlh3 can associate with nearby regions of dsDNA through a synapsis-type mechanism. In this case, the nucleoprotein conformation permits nicking activity to an interacting duplex in trans.

Our data indicate that Mlh1-Mlh3 is not creating a large number of nicks on a given DNA molecule, suggesting that an activated polymer may introduce as few as one nick per substrate. Our data do not give a clear understanding, however, of where within an active polymer a nick is created. In our assays, Mlh1-Mlh3 polymers bind and nick dsDNA nonspecifically, so mapping a nick to a unique heterodimer within a polymer is not feasible. One possibility is that an interior heterodimer within the polymer enjoys a high degree of stabilization and that there is a critical polymer length to optimize this stabilization, which triggers nicking. Introducing interruptions, such as insertion loops, interferes with achieving optimal stabilization. A second possibility is that the first Mlh1-Mlh3 molecule that binds DNA to initiate stable polymer formation introduces a nick. Mlh1-Mlh3 has a binding preference for branched DNA substrates and DNA with insertion loops. Incorporating features such as insertion loops into circular DNA inhibits Mlh1-Mlh3 activity, however. One interpretation of these data is that an Mlh1-Mlh3 polymer nucleates from the insertion loop because of it being a preferred binding site, but such binding distorts the conformation of the initial Mlh1-Mlh3 heterodimer so that it is inhibited from nicking. Our data in Fig 6 argue against this model, however. When wild-type and endonuclease-inactive proteins are mixed together at up to a 1:3 stoichiometric ratio, one would expect that if the initiating heterodimer were responsible for nicking, the presence of Mlh1-mlh3D523N would be inhibitory. We therefore favor a model in which an Mlh1-Mlh3 heterodimer interior to the polymer is activated and introduces nicks. The addition of other factors, such as Msh4-Msh5 or Exo1, may regulate the extent of polymerization that is required to induce Mlh1-Mlh3 nicking.

### Models to explain Mlh1-Mlh3 activity in vivo

How are dHJs resolved into COs in the Msh4-Msh5/Mlh1-Mlh3 pathway? Resolvases, such as Mus81-Mms4 and Yen1, which cleave dHJs in an Msh4-Msh5–independent pathway, produce both COs and NCOs, suggesting that they cleave each HJ in an independent manner. Such a mechanism does not necessitate a precise orientation of the endonuclease and only requires a nuclease to recognize and cleave DNA within a junction. In the Msh4-Msh5/Mlh1-Mlh3 pathway, only COs form as the result of dHJ resolution. Since Mlh1-Mlh3 is the major nuclease activity required in this pathway, either it is “presented” with an inherently asymmetric substrate or it must be organized in different orientations at each junction to facilitate asymmetric cleavage. The finding that in MMR the human MLH1-PMS2 endonuclease activity is strand-specifically activated by PCNA suggests that meiotic factors could regulate Mlh1-Mlh3 endonuclease specificity in an analogous way [49].

In meiosis, the two HJs present in a dHJ are not created simultaneously, and identical proteins may not occupy them prior to resolution. In the leptotene stage of meiotic prophase I, each DSB is resected to form two 3′ single-strand overhangs, one of which invades the homologous chromosome. The invading end is extended and stabilized by ZMM proteins, including Msh4-Msh5, in zygotene, creating the first HJ in the dHJ intermediate. In early pachytene, the newly synthesized invading strand can reanneal to the other side of the DSB in a process involving RPA, Rad52, Rad54, and potentially Zip3 to create the second HJ [16,50–58]. This process suggests how an asymmetry could be created between the two junctions to exclusively resolve dHJs into COs. It also implies a coordinated assembly of the dHJ substrate and that the junctions may be at least partially protected from Mus81-Mms4, which acts at the same time as Mlh1-Mlh3 during Meiosis I [25]. The complex assembly of the substrate and the in vivo requirements for other factors in creating and stabilizing the substrate strongly support the proposal that other protein factors, such as Msh4-Msh5 or other ZMM proteins, are present in precise positions prior to Mlh1-Mlh3’s activities and that these factors are likely critical for recruiting and orienting Mlh1-Mlh3. Msh4-Msh5 is explicitly implicated to function upstream of Mlh1-Mlh3 by meiotic crossover control experiments in which a *mms4Δ mlh1Δ* baker’s yeast strain exhibited a 13- to 15-fold decrease in crossing over compared to the wild type, but a *mms4Δ mlh1Δ msh5Δ* triple mutant only showed a 5-fold decrease, similar to the decrease observed in a *mms4Δ msh5Δ* double mutant [20]. We hypothesize, based on relative DNA-binding affinities between MLH proteins and MSH proteins, that once recruited, the Mlh1-Mlh3 polymer displaces Msh4-Msh5 [27,31,45] (Fig 10A and 10C). Such an idea is consistent with work in *Sordaria* in which Msh4 foci were observed to diminish between early and midpachytene, a time frame in which Mlh1-Mlh3 is believed to be recruited [8]. It is also supported by an experiment analogous to those shown in Fig 2 in which hydrolytically inactive *Eco*RI(E111Q) was tested as a roadblock for activation of the Mlh1-Mlh3 endonuclease. Although *Eco*RI(E111Q) bound to DNA, it did not have an inhibitory effect on Mlh1-Mlh3 activity (S7 Fig).

In addition to the ZMM and second-end capture factors, a candidate for an in vivo specificity factor is the nuclease-independent activity of Exo1, which was shown by Zakharyevich et al. to be required in conjunction with Mlh1-Mlh3 and Sgs1-Top3-Rmi1 in dHJ resolution [13,18]. It is known that Mlh1 physically interacts with Exo1 and that such an interaction may serve the purpose of stabilizing and thus activating Mlh1-Mlh3 on dHJ substrates in addition to interactions that are likely critical for MMR [59,60]. It is likely that in order to model HJ resolution by Mlh1-Mlh3 in vitro, these additional factors will need to be present and positioned appropriately. It is also likely that interactions with these other factors stabilize Mlh1-Mlh3 in such a way that a smaller amount of Mlh1-Mlh3 is required for activation than we can demonstrate in vitro in the absence of other factors. This is supported by the stimulation of Mlh1-Mlh3 by MMR factor Msh2-Msh3, in which the amount of nicked product observed with 100 nM Mlh1-Mlh3 can be observed with 25 nM Mlh1-Mlh3 in the presence of Msh2-Msh3 (S4 Fig; [26]).

Can we reconcile the Mlh1-Mlh3 enzymatic activities observed here with asymmetric resolution of dHJs? Endonuclease activity being contingent upon Mlh1-Mlh3 polymer formation would regulate the endonuclease activity in vivo and prevent promiscuous nicking of DNA substrates. In vivo, it is unlikely that there would be sufficient naked DNA to accommodate a polymer that can nick DNA at nonspecific sites as we observe in our in vitro assays. It would also restrain the double-strand break activity that we observe on very large substrates. During dHJ resolution, there are likely to be constraints imposed upon the endonuclease by both the substrate and other protein factors that tightly regulate the endonuclease activity and promote polymer formation and activation at specific sites. We also observe that disrupting the continuity of dsDNA has an inhibitory effect on endonuclease activity. This suggests that pure junction recognition, followed by polymer formation and activation, is unlikely to take place in vivo. It is likely that a very specific substrate and positioning of other factors, including Msh4-Msh5, provide a unique substrate for Mlh1-Mlh3 to act on (Fig 10C). In the absence of a fully reconstituted system, we provide early models for how dHJ resolution occurs in vivo. Below, we put forward possible models for dHJ resolution that exploit the biochemical properties presented here.

To our knowledge, there is no direct evidence that dHJs are covalently closed in vivo. If they are not, nicks would likely be present in dHJ structures at sites of DNA synthesis termination (Fig 10D, top). In such a scenario, an endonuclease recruited to a dHJ could nick the strand opposite to the preexisting nick present in an unligated HJ. Such a model has been previously suggested for a generic HJ resolvase activity [61]. One drawback of this model is that Mlh1-Mlh3 does not appear to recognize and cleave HJs like Mus81-Mms4. If DNA synthesis terminates within a junction, the resultant substrate is a nicked junction, which is recognized and cleaved efficiently by Mus81-Mms4. Thus, collaboration with a structure-selective endonuclease may be required (Fig 10D, right). If DNA synthesis terminates such that the nicks are some distance away from the junctions, an Mlh1-Mlh3 polymer could form and be directed to nick specifically upon encountering the unligated duplex (Fig 10D, left). Since DSBs are neither desired nor required for CO formation, this would necessitate an Mlh1-Mlh3 polymer to nick the duplex that does not contain the preexisting nick (nicking in trans). The ability of Mlh1-Mlh3 to nick in trans is supported by our observations in Fig 9. Such a model may also couple Mlh1-Mlh3 polymer directionality with DNA polarity, which we have not explicitly observed in vitro.

Modeling of recombination intermediates suggested that dHJs can isomerize between two different geometries (type I and II; [62]). Type I represents the archetypal depiction of a double Holliday junction (Fig 10E). Type II is an alternate configuration of a dHJ, which, like type I, involves a twisting or isomerization of one of the two HJs from a common intermediate ([62]; Fig 10E). The type II configuration provides an attractive model to integrate the Mlh1-Mlh3 biochemical activities described here; for resolution of this geometry into crossover products, cleavage can take place symmetrically by using an Mlh1-Mlh3 polymerization and nicking mechanism (Fig 10F; see below). A challenge in explaining how such geometries are relevant to recombination is why a type II configuration would be favored, as well as the steric issues associated with isomerization, which could include chromosome arm rotations.

Based on a molecular analysis of *zip1* mutants in yeast, Storlazzi et al. provide an elegant argument for the maintenance of a type II configuration [58]. They described an early role for Zip1 at the transition from resected DSBs to strand invasion steps: specifically, COs were significantly decreased in *zip1* mutants. Based on a mechanical stress model developed to explain CO patterning, they suggested that Zip1 acts in a CO differentiation pathway to preisomerize dHJs into type II junctions committed to a CO pathway. Such a model is attractive because it provides a way to deal with the steric issues outlined above. Consistent with this idea is a recent analysis in *Sordaria* of Hei10, a Zip3 family protein that has been implicated as a structure-based signaling molecule acting in several pathways, including meiotic recombination [42]. DeMuyt et al. observed, using three-dimensional structured illumination microscopy, that ~50% of Hei10 foci localize to sites of twists in the synaptonemal complex (SC) [42]. The authors argue that “this structural distortion points to structural and/or geometric interplay between the DNA events of CO formation and the SC.” This analysis may also be consistent with work performed by Oke et al. in observing recombination products in yeast, wherein the authors suggest that Zip3 is required for the conversion of dHJs into exclusively CO products [51]. To this point, if we invoke the presence of a type II double Holliday junction substrate in CO formation, one can imagine Mlh1-Mlh3 being recruited and forming a polymer to cut at both junctions (Fig 10F). Perhaps between the two junctions there is inadequate DNA length to accommodate the critical length of an Mlh1-Mlh3 polymer.

In budding yeast, the two junctions of a dHJ have been estimated to be either a few hundred base pairs away from each other [63,64] or converged to point junctions [64]. The requirement for a helicase/topoisomerase (Sgs1-Top3-Rmi1) in the Msh4-Msh5/Mlh1-Mlh3 pathway also suggests that junctions may converge or nearly converge, creating a structure that Mlh1-Mlh3 is capable of cleaving [21,23]. Such a mechanism would also require other factors to constrain Mlh1-Mlh3 polymer formation so that nicking can be localized. It should be noted, however, that nicking does not need to explicitly take place at a HJ to yield a CO product. Nicks could be produced away from the junction. A subsequent branch migration step would then be sufficient for resolution.

Our data in Fig 9 support the idea that Mlh1-Mlh3 can interact with two DNA molecules simultaneously and that these interactions can stimulate the endonuclease to act on a substrate that it does not act on in the absence of the second substrate. These data in conjunction with observations for Mlh1-Pms1 presented by Hall et al. [31] support a synapsis-type mechanism. Such an activity could be used in meiosis to permit nicking to interacting duplex regions in trans. Orientation of Mlh1-Mlh3 by other factors could aid in directing the polymer to nick so that CO products are generated.

### How might Mlh1-Mlh3 act in MMR?

Mlh1-Mlh3 is a member of the MLH family of MMR proteins and plays a minor role in repairing mismatches recognized by Msh2-Msh3 [3,4]. Models for MMR in eukaryotes suggest that mismatches are recognized by MSH factors, which recruit MLH factors to specifically cleave the newly replicated strand. The endonuclease activity of Mlh1-Pms1 (MLH1-PMS2 in humans) is activated through interactions with the DNA replication processivity clamp PCNA [28,29]. Pluciennik et al. propose that asymmetric loading of PCNA is responsible for directing the Mlh1-Pms1 endonuclease to cleave the mismatch-containing strand [49]. Together, these observations support the idea that MLH endonuclease activities are directed by other factors and are consistent with the biochemical studies of Mlh1-Mlh3 presented here. Interestingly, work by the Crouse laboratory suggests that Mlh1-Mlh3 acts in conjunction with Mlh1-Pms1 during MMR [4]. Mlh1-Mlh3 appears to lack residues present in other MutL proteins that appear critical for activation by the DNA replication processivity clamp [65]; consistent with this, Mlh1-Mlh3 endonuclease is not activated by RFC and PCNA in vitro [26,27]. These observations suggest that during MMR, Mlh1-Mlh3 is recruited and activated by Msh2-Msh3 but must retain an intimate association with Mlh1-Pms1, which is presumably oriented by PCNA, to coordinate MLH endonuclease activities with strand-specific repair.

## Materials and methods

### Oligonucleotides

Sequences of all oligonucleotides used in this study are listed in S1 Table.

For experiments in Fig 1, homoduplex and +8 loop substrates were constructed from AO3142 and either AO3144 (homoduplex) or AO3143 (+8) as described [26,45]. HJ substrate X26 [66], with a 26-bp homologous core allowing branch migration, was constructed from AO3147 (X26-1), AO3148 (X26-2), AO3149 (X26-3), and AO3150 (X26-4). Annealed substrates were purified by HR S-300 spin columns (GE Healthcare).

### Protein purification and preparation

Yeast wild-type Mlh1-Mlh3 and Mlh1-mlh3D523N were purified from baculovirus-infected *Sf*9 insect cells as previously described [26]. Human MLH1-PMS2 was a gift from Peggy Hsieh’s laboratory and was purified as previously described [67,68]. Yeast Mlh1-Pms1, Msh2-Msh3, RFC, and PCNA were purified from yeast as described previously [69–72].

For experiments in S1 Fig, either 200 units of lambda protein phosphatase (NEB) or 50 units of CDK1-cyclinB (NEB) was combined with purified Mlh1-Mlh3 (1.2 μM final concentration) in the supplied buffer in a 15 μL reaction. The reaction was allowed to proceed according to the manufacturer’s instructions. For CDK1-cyclinB, we performed a control with Mlh1-Mlh3 and Ɣ ^32^-ATP and with Ɣ ^32^-ATP alone, followed by loading onto a Bio-Rad P-30 spin column. After centrifugation, Mlh1-Mlh3 protein elutes from the column, but Ɣ ^32^-ATP is retained. The specific activity of the probe was 30 CPM/fmol. Approximately 16,000 fmol of Mlh1-Mlh3 was reacted with ~20,000 fmol of Ɣ ^32^-ATP (conditions reported in the Materials and methods; except for the assayed complex, no radioactivity was used, and a larger excess of ATP was included). For the reaction that did not contain Mlh1-Mlh3, the elutant did not contain radioactive signal above background (as measured by a Geiger counter). For the reaction that contained Mlh1-Mlh3, the elutant was radioactive (~500,000 CPM, which is equivalent to ~16,000 fmol of material given the specific activity of the probe), indicating that a majority of the protein was radiolabeled if only one site per complex was phosphorylated.

With respect to the lambda protein phosphatase experiments, we treated the radiolabeled Mlh1-Mlh3 above with lambda phosphatase. After the spin column step, the eluant no longer gave a radioactive signal above background, as measured using a Geiger counter. We also performed a spectroscopic control using *p*-nitrophenyl phosphate using the same conditions used to treat Mlh1-Mlh3. Those conditions converted 100% of the *p*-nitrophenyl phosphate to *p*-nitrophenol, which is detected at 405 nm and has an extinction coefficient of 18,000 M^-1^cm^-1^. We also ran an SDS-PAGE on Mlh1-Mlh3 samples treated with lambda protein phosphatase and did not observe a mobility shift, which suggests that the complex is at least not hyperphosphorylated.

### Circular substrates

The synthesized 7.2 kb circular DNA substrates in Fig 3 and S4 Fig were generated essentially by using the protocol described by Baerenfaller et al. [35]. HPLC-purified primers were obtained from IDT. Homoduplex 7.2 kb circular substrate was generated from AO3266 and biotinylated 7.2 kb circular substrate was generated from BIO_M13mp18. +8 loop–containing 7.2 kb circular substrate was generated from AO3267 (with loop mismatch disrupting *Bmr*I restriction site). Primers were phosphorylated on their 5′ ends by T4 polynucleotide kinase (NEB) in a 50 μL reaction in the supplied buffer containing 6 μM primer, 1 mM ATP, and 10 units enzyme at 37°C for 60 min. Kinase was inactivated for 20 min at 65°C. Unincorporated nucleotide was removed using a P-30 spin column (Bio-Rad) according to the manufacturer’s instructions. Phosphorylated primer (1 μM) was annealed to 250 nM M13mp18 ssDNA template (Affymetrix) in a 25 μL reaction in a buffer containing 50 mM Tris-HCl (pH 7.5), 10 mM MgCl_2_, and 10 mM DTT by incubating for 6 min at 85°C then cooling to room temperature overnight. Annealed primers were extended and closed circular substrates were generated by incubation in a 50 μL reaction containing 96 nM primer template with 1 mM dNTPs, 30 units T4 DNA polymerase (NEB), and 1,000 units T4 DNA ligase (NEB) in a buffer containing 50 mM Tris-HCl (pH 7.5), 100 μg/mL BSA, 10 mM MgCl_2_, 10 mM DTT, and 1 mM ATP. Reactions were incubated at 37°C for 60 min, followed by enzyme inactivation at 70°C for 20 min. Closed circular DNA was isolated from ssDNA and open circle DNA by resolution in a 0.8% agarose gel and extraction using a QIAGEN gel extraction kit. The presence of the +8 loop mismatch was verified by sequencing and lack of *Bmr*I restriction enzyme digestion. Radiolabeled circular substrate used in S4 Fig was prepared by an identical method to the above with the exception that primers AO3266, AO3267, or AO3346 (for generating substrate in which the radiolabel is on the strand opposing that containing the mismatch) were phosphorylated by incubation with Ɣ^32^-ATP (Perkin Elmer) prior to annealing.

For experiments in Fig 2C, S2 Fig, Fig 7, and S5 Fig, pUC18 2.7 kb and pBR322 4.4 kb closed circular plasmids were purchased from Invitrogen. The 7 (pEAE399), 12 (pEAE99, pEAO202, pEAE324), 14 (pEAE107), and 15 (pEAM58) kb plasmids were amplified and mini-prepped from DH5α-competent cells by standard methods. Prenicked circular substrates used in Fig 8 were generated as previously described using *Nt*.*Bsp*QI, *Nt*.*Bst*NBI, or *Nt*.*Alw*I purchased from New England Biolabs [26].

### Large linear substrates

The 2.7, 7 kb, and 12 kb linear DNA substrates were generated by digesting pUC18, pEAE399, or pEAE99 with *Hin*dIII (NEB) according to the manufacturer’s instructions. Reactions were incubated at 37°C for 60 min, followed by enzyme inactivation at 80°C for 20 min. Linearized fragment was isolated via resolution by agarose gel and gel extraction (QIAGEN). Biotinylated pUC18 used in Fig 3 was prepared by incubating the *Hin*dIII fragment with 1 mM dATP, dCTP, biotin-11-dGTP (Perkin Elmer), and dTTP and 10 units of *Bsu* DNA polymerase large fragment (NEB) in the supplied buffer at 37°C for 60 min, followed by enzyme inactivation at 75°C for 20 min. Excess nucleotide was removed using a P-30 spin column (Bio-Rad) according to the manufacturer’s instructions. Streptavidin was bound to biotinylated DNA by incubating 50 nM DNA with 1 μM streptavidin (NEB) at room temperature in the endonuclease reaction buffer for 15 min immediately prior to use. The presence of streptavidin was confirmed by gel shift.

Substrates used in Fig 3E were generated identically to those used in Fig 3C to created circularized versions. The homoduplex or +8 loop mismatch–containing substrate was then linearized with the indicated restriction enzyme (NEB) according to the manufacturer’s instructions. Linearized DNA was then gel isolated to be used in endonuclease assays.

For radiolabeled linear substrates used in filter binding reactions, pUC18, pEAE399, or pEAE99 *Hin*dIII fragments were extended by *Bsu* DNA polymerase large fragment using α ^32^-dATP by a method analogous to that used to biotinylate linear DNA fragment above.

### Endonuclease assays

Endonuclease reactions were performed as previously described [26] in endonuclease buffer: 20 mM HEPES- KOH (pH 7.5), 20 mM KCl, 0.2 mg/mL BSA, 1% glycerol, and 1 mM MgCl_2_ unless otherwise indicated. Reactions were stopped by the addition of a stop mix solution containing final concentrations of 0.1% SDS, 14 mM EDTA, and 0.1 mg/mL ProteinaseK (NEB). Products were resolved by 1% agarose gel containing 0.1 μg/mL ethidium bromide, which results in covalently closed circular DNA isoforms migrating similarly to supercoiled DNA. Gels were run in 1x TAE (Tris-acetate-EDTA; 40 mM Tris base, 20 mM acetic acid, 1 mM EDTA) at 100 V for 40 min or by denaturing agarose gel where indicated. For experiments with circular DNA substrate analyzed by denaturing agarose gel, circular DNA was linearized with *Hin*dIII for 60 min after incubation with Mlh1-Mlh3 but prior to the addition of stop mix. Linear substrate was treated identically. Denaturing agarose gels consist of 1% (w/v) agarose, 30 mM NaCl, 2 mM EDTA (pH 7.5) run in a buffer containing 30 mM NaOH and 2 mM EDTA. Prior to sample loading, reactions were diluted in five volumes of buffer containing 180 mM NaOH, 6 mM EDTA, 20% glycerol, 0.1% xylene cyanol, and 0.1% bromophenol blue, heated for 5 min at 70°C, then cooled for 3 min on ice. Gels were run at 50 V for ~3 h. After running, alkaline agarose gels were neutralized in 0.5 M Tris-HCl (pH 7.5) for 30 min and stained with 0.5 μg/mL ethidium bromide for ~2 h. To generate a marker for closed circular, linear, and nicked 12 kb plasmid, pEAE99 was treated for 2 min with 0.1 μL of a 1:1,000 dilution of DNaseI (NEB) at 37°C. Quantifications were performed using GelEval (FrogDance Software, v1.37), and negative control reactions were used for background subtractions.

### Mapping Mlh1-Mlh3–generated nicks opposite preexisting nicks

pUC18 plasmid was either nicked with *Nt*.*Bsp*QI or linearized using *Sap*I (NEB) according to the manufacturer’s instructions with heat inactivation and gel isolation of the nicked or linear product. Nicked plasmid was then used as an endonuclease substrate in a reaction performed with 24 replicates containing 300 nM Mlh1-Mlh3. After stopping the reactions, replicates were combined and the DNA was ethanol precipitated by standard methods. The concentrated DNA sample was then resolved by 0.8% agarose gel, and the linear endonuclease product was gel isolated. This product or control linearized plasmid (5 nM) was annealed to the radiolabeled primer (50 nM) either complementary to the strand with the *Nt*.*Bsp*QI nicking site (primer A [AO3516]) or to the primer complementary to the opposing strand (primer B [AO3518]). Template annealed to the radiolabeled primer was then isolated using S-300 HR spin columns (GE) and incubated with 2.5 mM dNTPs and 2,000 U of T4 DNA polymerase (NEB). Primer extension products were analyzed by 15%, 8 M urea PAGE and phosphorimaging. Radiolabeled oligonucleotide AO3535 was used as a marker for migration of a 60-mer primer extension product.

### Filter binding

DNA filter binding assays were performed essentially as described [73]. Briefly, 20 μL reactions containing 15 μM total nucleotide were combined with increasing amounts of protein in a reaction containing 20 mM Tris-HCl (pH 7.5), 0.01 mM EDTA, 2 mM MgCl_2_, 40 μg/mL BSA, and 0.1 mM DTT. Upon the addition of Mlh1-Mlh3, reactions were incubated for 10 min at 30°C. The reaction was then filtered through KOH-treated nitrocellulose filters using a Hoefer FH225V filtration device for approximately 1 min. Filters were subsequently analyzed by scintillation counting.

### Electron microscopy data acquisition and image processing

Mixtures of Mlh1-Mlh3 alone or in combination with either circular or linear DNA were applied at the concentrations stated in the Results onto EM grids freshly coated with a continuous layer of amorphous carbon. Grids were floated on a 5 μL drop of the diluted assembly reaction for 2 min immediately after a glow discharge treatment of 5 mA for 15 s. Excess of sample was blotted with filter paper and the grids were stained with 1% uranyl acetate for 1 min. Grids were loaded in a room-temperature holder and introduced into a FEI Tecnai F20 electron microscope operated at 200 kV and equipped with a Gatan K2 Summit direct detector camera. This detector was used in counting movie mode with five electrons per pixel per second for 15-s exposures and 0.5 s per frame. This method produced movies consisting of 30 frames with an exposure rate of ~1 e^-^/Å^2^. Movies were collected with a defocus range of –1 to –2.5 microns and a nominal magnification of 11,500x, which produced images with a calibrated pixel size of 3.15Å. The 30 frames in each move were aligned using the program alignframesleastsquares_list [74] and averaged into one single micrograph with the shiftframes_list program [74]. These programs are available from (https://sites.google.com/site/rubinsteingroup/home). These programs perform whole frame alignment of the movies using previously published motion correction algorithms [75]. A denoising filter using Photoshop was applied to the entire image to obtain the figures shown.

### Radiolabeled proximity assay

For experiments in S4 Fig, circular substrate with and without a mismatch was synthesized as described above except with the inclusion of a radioactive phosphate on the 5′-end of the primer used for synthesis. When Msh2-Msh3 was present, the protein was preincubated with the substrate at 30°C for 10 min, after which 20 nM Mlh1-Mlh3 (final concentration) was added to reactions. The reaction was then incubated, stopped, and deproteinated as described above. The reaction was then either resolved by agarose gel to determine the total amount of nicking, or the DNA was digested with *Bsa*HI and *Bsr*GI at 37°C for 60 min. The digested sample was then resolved by 4%, 8 M urea PAGE and phosphorimaged.

### Religation experiments

The closed circular form of pEAE99 was initially gel isolated and treated with *ScaI*, *Hin*dIII, or used as a substrate in an Mlh1-Mlh3 endonuclease reaction containing 300 nM Mlh1-Mlh3 in quintuplicate. The linearized product was then gel isolated from these reactions and replicates were combined. Where + T4 polymerase is indicated, in a 10 μL reaction, ~1 μg of linearized DNA was combined with 100 μM dNTPs in NEB buffer 2.1. One unit of T4 DNA polymerase (NEB) was added to the reaction. The reaction was incubated for 15 min at 12°C followed by a heat inactivation step at 75°C for 20 min. An 8 μL portion of the reaction was then added to a 10 μL reaction containing the supplied buffer and 400 units of T4 DNA ligase (NEB) where indicated. The reaction was incubated at room temperature for 2 h followed by heat inactivation per the manufacturer’s instructions.

For all plotted data, individual data points from each trial as well as means and standard deviations are found in S1 Data.

## Supporting information

S1 Fig Neither treatment with phosphatase nor treatment with CDK1 affect endonuclease activity. (A) *Left*, SDS-PAGE analysis of treatment of 1.2 μM Mlh1-Mlh3 with 200 U lambda (λ) protein phosphatase (NEB) (see Materials and methods). Where - λ PP is indicated, a mock treatment was performed omitting the phosphatase. Where + is indicated, 200 U of phosphatase was added to the reaction. No gel shift was observed. *Right*, agarose gel analyzing nicking on 2.7 kb circular substrate following treatment of 1.2 μM Mlh1-Mlh3 with 200 U lambda (λ)protein phosphatase. Where - Mlh1-Mlh3 is indicated, a mock treatment was performed omitting Mlh1-Mlh3 and combining with DNA in the endonuclease reaction to assess any background nicking from increasing amounts of the phosphatase treatment conditions. Where - phosphatase is indicated, Mlh1-Mlh3 was subjected to treatment with the phosphatase buffer, but MnCl_2_ and phosphatase were omitted. + phosphatase is the full reaction including phosphatase and Mlh1-Mlh3. (B) Average of quantification for four separate experiments, errors bars represent standard deviation. (C) Agarose gel analyzing nicking on 2.7 kb circular substrate following treatment of 1.2 μM Mlh1-Mlh3 with 50 U CDK1-cyclinB (NEB) (see Materials and methods). Where - Mlh1-Mlh3is indicated, a mock treatment was performed omitting Mlh1-Mlh3 similar to that described above. Where - kinase is indicated, Mlh1-Mlh3 was subjected to treatment with the CDK1 buffer, but CDK1 was omitted. + kinase is the full reaction including CDK1 and Mlh1-Mlh3. (D) Average of quantification for three separate experiments, errors bars represent standard deviation.
(TIFF)

S2 Fig Yeast Mlh1-Mlh3 has identical behavior using Mn^2+^ to activate the endonuclease. (A) Denaturing agarose analysis of yeast Mlh1-Mlh3 nicking on circular pUC18 (2.7 kb) (black) or *Hin*dIII linearized pUC18 (red) in the presence of 1 mM MnSO_4_. (B) Quantification of data in A; fraction nicked defined as fraction of substrate lost plotted against yeast Mlh1-Mlh3 concentration. (C) Native agarose gel electrophoresis analysis of yeast Mlh1-Mlh3 (150 nM) endonuclease activity on circular substrate ranging from 2.7 kb to 12 kb. The concentration of nucleotide in each reaction is 15 μM. Lane 1 contains 2-log DNA ladder (NEB). Where + Mn^2+^is indicated, 1 mM MnSO_4_ was added to the reaction. (D) Quantification of nicking in lanes 4, 7, 10, and 13 in C averaged from two separate experiments. Error bars indicate standard deviation.
(TIFF)

S3 Fig Mlh1-Mlh3 displays similar nicking capabilities on negatively supercoiled and relaxed circular DNA. Relaxed circular pUC18 plasmid was prepared by linearization with *Hin*dIII, followed by treatment with T4 DNA ligase (NEB). Ligated product and supercoiled pUC18 were resolved in one-half of an agarose gel not containing ethidium bromide. In the other half of the gel, *Nt*.*Bst*NBI-digested pUC18 and supercoiled pUC18 were resolved as markers. After running, the gel was cut and the half containing the markers was stained with ethidium bromide. The relaxed circlular and supercoiled circluar substrates were extracted from the unstained half using the stained half as a guide. Endonuclease reactions were assembled and carried out as described in the *Materials and Methods* using either supercoiled or relaxed circular DNA as a substrate. *Left*, where + Mlh1-Mlh3 is indicated, reactions contain 300 nM Mlh1-Mlh3. In lanes 4–7 and 10–13, Mlh1-Mlh3 is 50, 150, 300, and 500 nM, respectively. *Right*, quantification of agarose gel.
(TIFF)

S4 Fig Mlh1-Mlh3 nicking is non-specific on 7.2 kb DNA with and without a +8 loop mismatch and with and without Msh2-Msh3. (A) Endonuclease activity performed with 25 nM wild-type or D523N Mlh1-Mlh3 in the presence of 60 nM Msh2-Msh3 and/or 0.5 mM ATP where indicated. (B) Schematic describing mapping assay. Radiolabeled substrate combined with 40 nM Msh2-Msh3 and 20 nM Mlh1-Mlh3. After endonuclease activity was stopped, substrate was cleaved with *Bsa*HI and *Bsr*GI (2.3 kb fragment) for analysis by denaturing PAGE. (C) *Left*, total nicking on circular substrate measured by agarose gel analysis. *Right*, 8 M urea PAGE analysis of Mlh1-Mlh3 nicking ± Msh2-Msh3. Amount of radioactive probe was quantified and the fraction of substrate nicked was calculated as amount of radioactivity lost compared to the negative controls (lanes 1, 4, and 7). < indicates that the amount of substrate lost was less than 3%. Approximate position of 150 nucleotide migration is indicated.
(TIFF)

S5 Fig Endonuclease experiments with human MLH1-PMS2. All reactions contain 1 μM yeast RFC and yeast PCNA, 0.5 mM ATP, and 1 mM Mn^2+^ unless otherwise indicated. (A) 15 μM total nucleotide in each reaction combined with 150 nM human MLH1-PMS2 in the presence of RFC/PCNA with ATP. Experiment is otherwise identical to that performed in Fig 2C. (B) Quantification of data in A combined with data from an identical experiment using a 1.4 kb and 15 kb circular substrate. 2.7, 7.2, and 12 kb substrates are as described in the *Materials and Methods*. 1.4 kb circular substrate was generated by re-ligating the ~1400 bp *Bss*SαI fragment of pUC18. 15 kb circular substrate is plasmid pEAM58 amplified and mini-prepped from DH5α competent cells by standard methods. Reactions were combined as described in A and in Fig 2C for human MLH-PMS2 and yeast Mlh1-Mlh3. (C) Denaturing agarose analysis of human MLH1-PMS2 nicking on circular pUC18 (2.7 kb) (black) and linearized pUC18 (red). (D) Average of two separate experiments as shown in C. (E) Human MLH1-PMS2 endonuclease activity on a 2.7 kb circular DNA substrate is inhibited by pre-incubating human MLH1-PMS2 with oligonucleotide substrates. 50 nM human MLH1-PMS2 was pre-incubated with increasing amounts of ~50 bp double stranded oligonucleotide substrates for 15 minutes at 30°C (0–2000 nM), either homoduplex or substrate with a +8 loop. After the pre-incubation step, reactions were challenged with 3.6 nM 2.7 kb circular substrate and incubated by conditions described for endonuclease assays in the *Materials and Methods* and analyzed by agarose gel. (F) Average of quantification for four separate experiments from E, errors bars represent standard deviation. (G) hMLH1-PMS2 (300 nM) does not create linear product on 2.7 kb closed or nicked circular substrate. Reaction was performed as described for Fig 8A and 8B.
(TIFF)

S6 Fig Mlh1-Mlh3 creates linear product on plasmid substrates 12 kb or larger. (A-B) Experiment performed identical to that in Fig 2C using the indicated sized plasmids as substrates. Sequences of 12 kb plasmids differ from that used in Figs 2C and 7.
(TIFF)

S7 Fig Mlh1-Mlh3 is not inhibited by EcoRI(E111Q). *Eco*RI(E111Q) is a variant of *Eco*RI that binds to, but does not cleave, the *Eco*RI recognition sequence [76]. In a 5 μL reaction, 3.2 μM of this variant was combined with 40 μM (concentration of nucleotide) DNA substrate (either 2.7 kb closed circular or 7.2 kb closed circular; each of which have one *Eco*RI site) in the Mlh1-Mlh3 endonuclease reaction buffer and incubated for 30 min at 37°C. Binding was confirmed by gel shift. *Eco*RI(E111Q)-bound substrate or substrate without *Eco*RI(E111Q) was then combined in an endonuclease reaction (15 μM final nucleotide concentration) with increasing amounts of Mlh1-Mlh3. The reaction was combined, allowed to proceed, and stopped as described in the *Materials and Methods*. Reactions were analyzed by agarose gel and quantified.
(TIFF)

S1 Table Oligonucleotides used in this study. (DOCX)

S1 Data Individual data points, means, and standard deviations. (XLSX)

## Abbreviations

cc: closed circular substrate
CO: crossover
dHJ: double Holliday junction
DSB: double-strand break
dsDNA: double-stranded DNA
EDTA: ethylenediaminetetraacetic acid
EMSA: electrophoretic mobility shift assay
HJ: Holliday junction
KCl: potassium chloride
MLH: MutL homolog
MMR: mismatch repair
MSH: MutS homolog
n: nicked product
NCO: noncrossover
PAGE: polyacrylamide gel electrophoresis
SA: streptavidin
SC: synaptonemal complex
SDS: sodium dodecyl sulfate
SEI: single-end invasion
TopIB: type IB DNA topoisomerase
