# Supplementary figures and images for: The mismatch repair and meiotic recombination endonuclease Mlh1-Mlh3 is activated by polymer formation and can cleave DNA substrates in trans
Source: PLoS Biol. 2017 Apr 28;15(4):e2001164. doi: 10.1371/journal.pbio.2001164 (PMC5409509; doi:10.1371/journal.pbio.2001164)

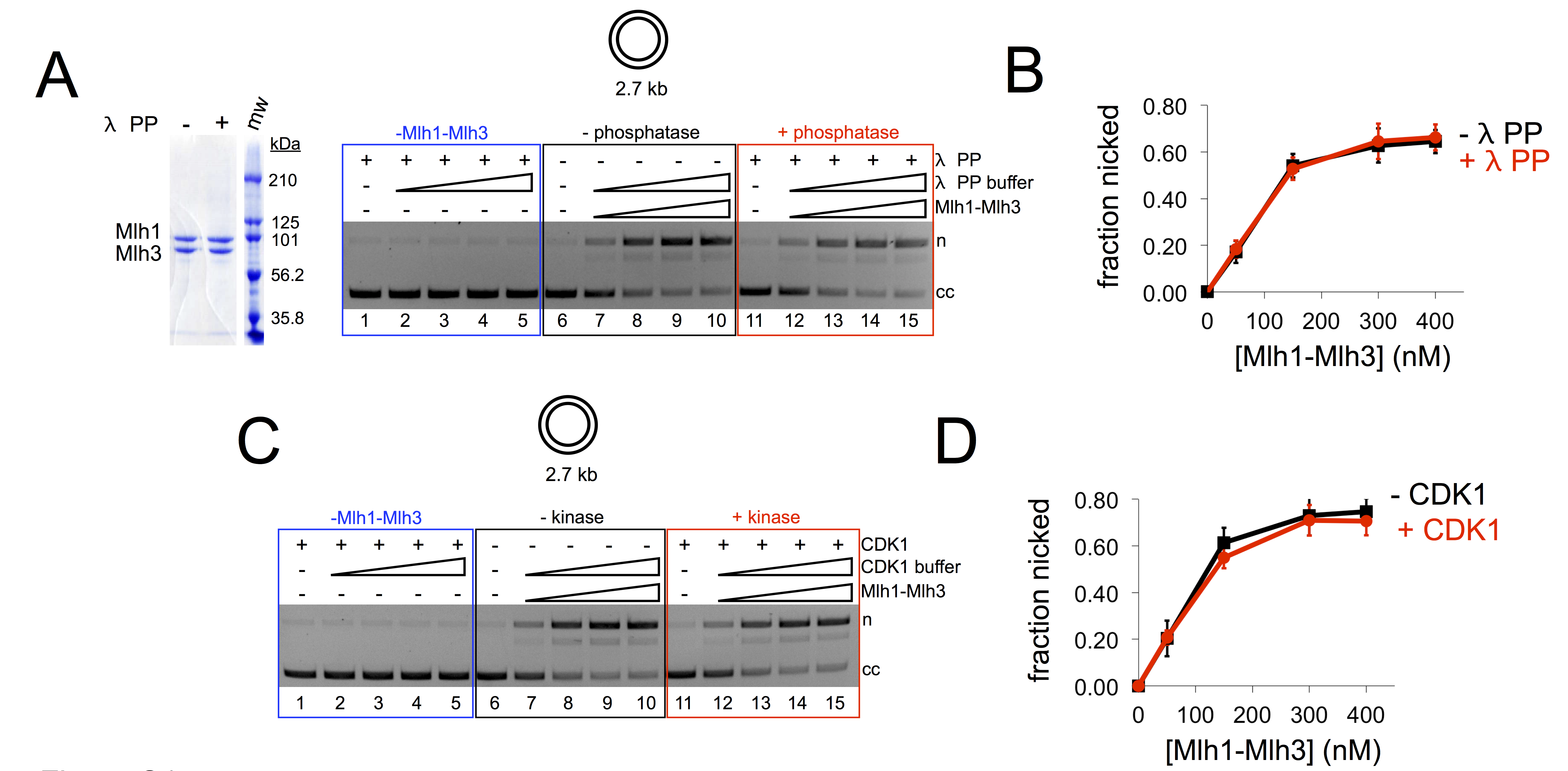

Supplement: S1 Fig — (A) Left, SDS-PAGE analysis of treatment of 1.2 μM Mlh1-Mlh3 with 200 U lambda (λ) protein phosphatase (NEB) (see Materials and methods). Where - λ PP is indicated, a mock treatment was performed omitting the phosphatase. Where + is indicated, 200 U of phosphatase was added to the reaction. No gel shift was observed. Right, agarose gel analyzing nicking on 2.7 kb circular substrate following treatment of 1.2 μM Mlh1-Mlh3 with 200 U lambda (λ)protein phosphatase. Where - Mlh1-Mlh3 is indicated, a mock treatment was performed omitting Mlh1-Mlh3 and combining with DNA in the endonuclease reaction to assess any background nicking from increasing amounts of the phosphatase treatment conditions. Where - phosphatase is indicated, Mlh1-Mlh3 was subjected to treatment with the phosphatase buffer, but MnCl2 and phosphatase were omitted. + phosphatase is the full reaction including phosphatase and Mlh1-Mlh3. (B) Average of quantification for four separate experiments, errors bars represent standard deviation. (C) Agarose gel analyzing nicking on 2.7 kb circular substrate following treatment of 1.2 μM Mlh1-Mlh3 with 50 U CDK1-cyclinB (NEB) (see Materials and methods). Where - Mlh1-Mlh3is indicated, a mock treatment was performed omitting Mlh1-Mlh3 similar to that described above. Where - kinase is indicated, Mlh1-Mlh3 was subjected to treatment with the CDK1 buffer, but CDK1 was omitted. + kinase is the full reaction including CDK1 and Mlh1-Mlh3. (D) Average of quantification for three separate experiments, errors bars represent standard deviation. (TIFF) [file pbio.2001164.s001.tiff]

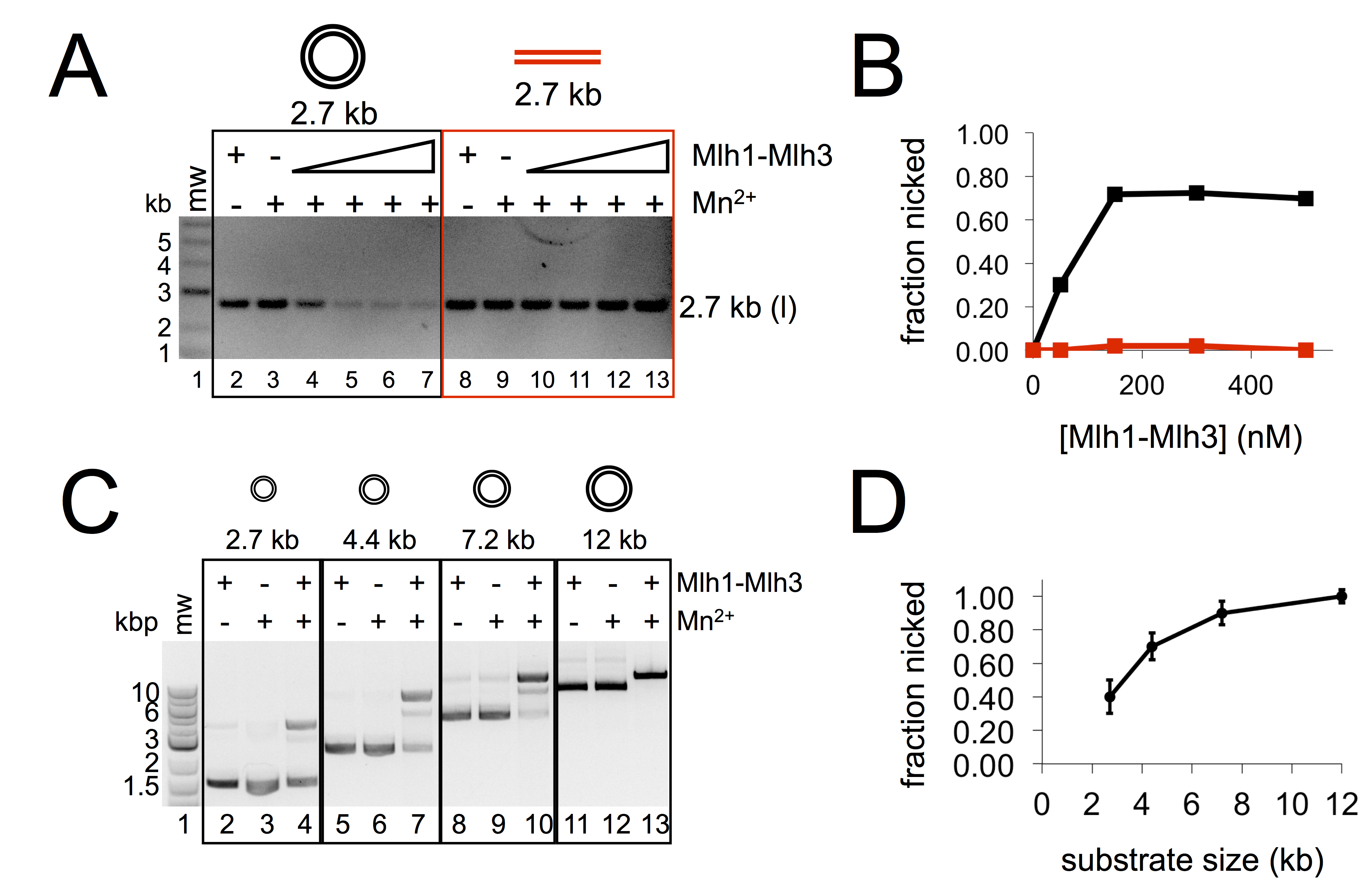

Supplement: S2 Fig — (A) Denaturing agarose analysis of yeast Mlh1-Mlh3 nicking on circular pUC18 (2.7 kb) (black) or HindIII linearized pUC18 (red) in the presence of 1 mM MnSO4. (B) Quantification of data in A; fraction nicked defined as fraction of substrate lost plotted against yeast Mlh1-Mlh3 concentration. (C) Native agarose gel electrophoresis analysis of yeast Mlh1-Mlh3 (150 nM) endonuclease activity on circular substrate ranging from 2.7 kb to 12 kb. The concentration of nucleotide in each reaction is 15 μM. Lane 1 contains 2-log DNA ladder (NEB). Where + Mn2+is indicated, 1 mM MnSO4 was added to the reaction. (D) Quantification of nicking in lanes 4, 7, 10, and 13 in C averaged from two separate experiments. Error bars indicate standard deviation. (TIFF) [file pbio.2001164.s002.tiff]

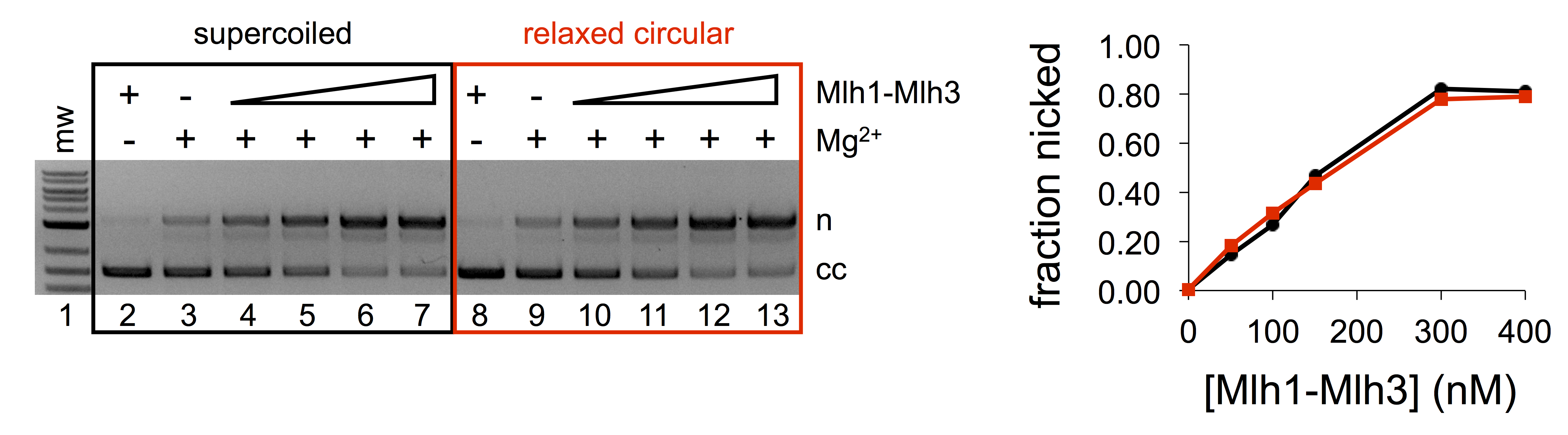

Supplement: S3 Fig — Relaxed circular pUC18 plasmid was prepared by linearization with HindIII, followed by treatment with T4 DNA ligase (NEB). Ligated product and supercoiled pUC18 were resolved in one-half of an agarose gel not containing ethidium bromide. In the other half of the gel, Nt.BstNBI-digested pUC18 and supercoiled pUC18 were resolved as markers. After running, the gel was cut and the half containing the markers was stained with ethidium bromide. The relaxed circlular and supercoiled circluar substrates were extracted from the unstained half using the stained half as a guide. Endonuclease reactions were assembled and carried out as described in the Materials and Methods using either supercoiled or relaxed circular DNA as a substrate. Left, where + Mlh1-Mlh3 is indicated, reactions contain 300 nM Mlh1-Mlh3. In lanes 4–7 and 10–13, Mlh1-Mlh3 is 50, 150, 300, and 500 nM, respectively. Right, quantification of agarose gel. (TIFF) [file pbio.2001164.s003.tiff]

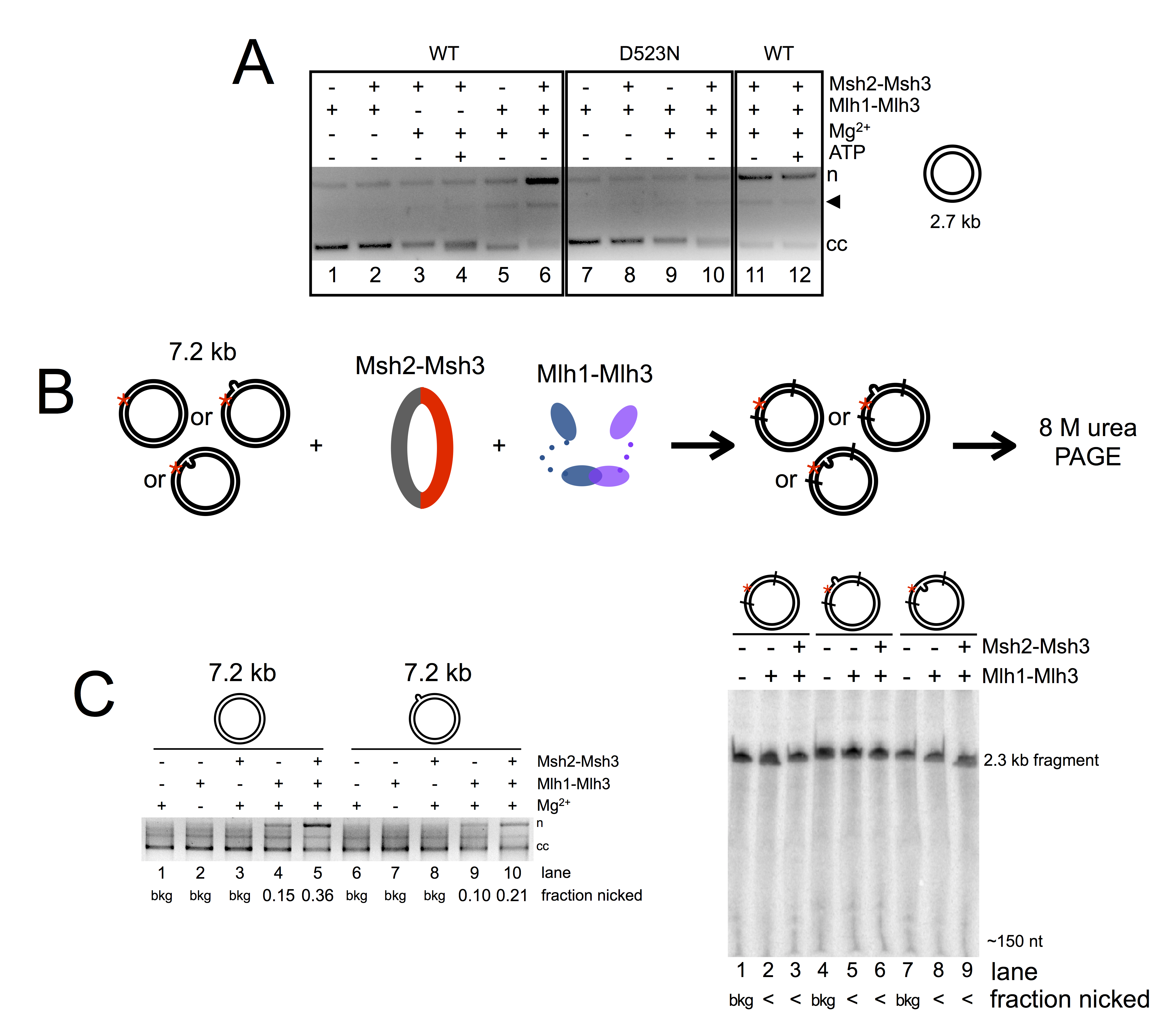

Supplement: S4 Fig — (A) Endonuclease activity performed with 25 nM wild-type or D523N Mlh1-Mlh3 in the presence of 60 nM Msh2-Msh3 and/or 0.5 mM ATP where indicated. (B) Schematic describing mapping assay. Radiolabeled substrate combined with 40 nM Msh2-Msh3 and 20 nM Mlh1-Mlh3. After endonuclease activity was stopped, substrate was cleaved with BsaHI and BsrGI (2.3 kb fragment) for analysis by denaturing PAGE. (C) Left, total nicking on circular substrate measured by agarose gel analysis. Right, 8 M urea PAGE analysis of Mlh1-Mlh3 nicking ± Msh2-Msh3. Amount of radioactive probe was quantified and the fraction of substrate nicked was calculated as amount of radioactivity lost compared to the negative controls (lanes 1, 4, and 7). < indicates that the amount of substrate lost was less than 3%. Approximate position of 150 nucleotide migration is indicated. (TIFF) [file pbio.2001164.s004.tiff]

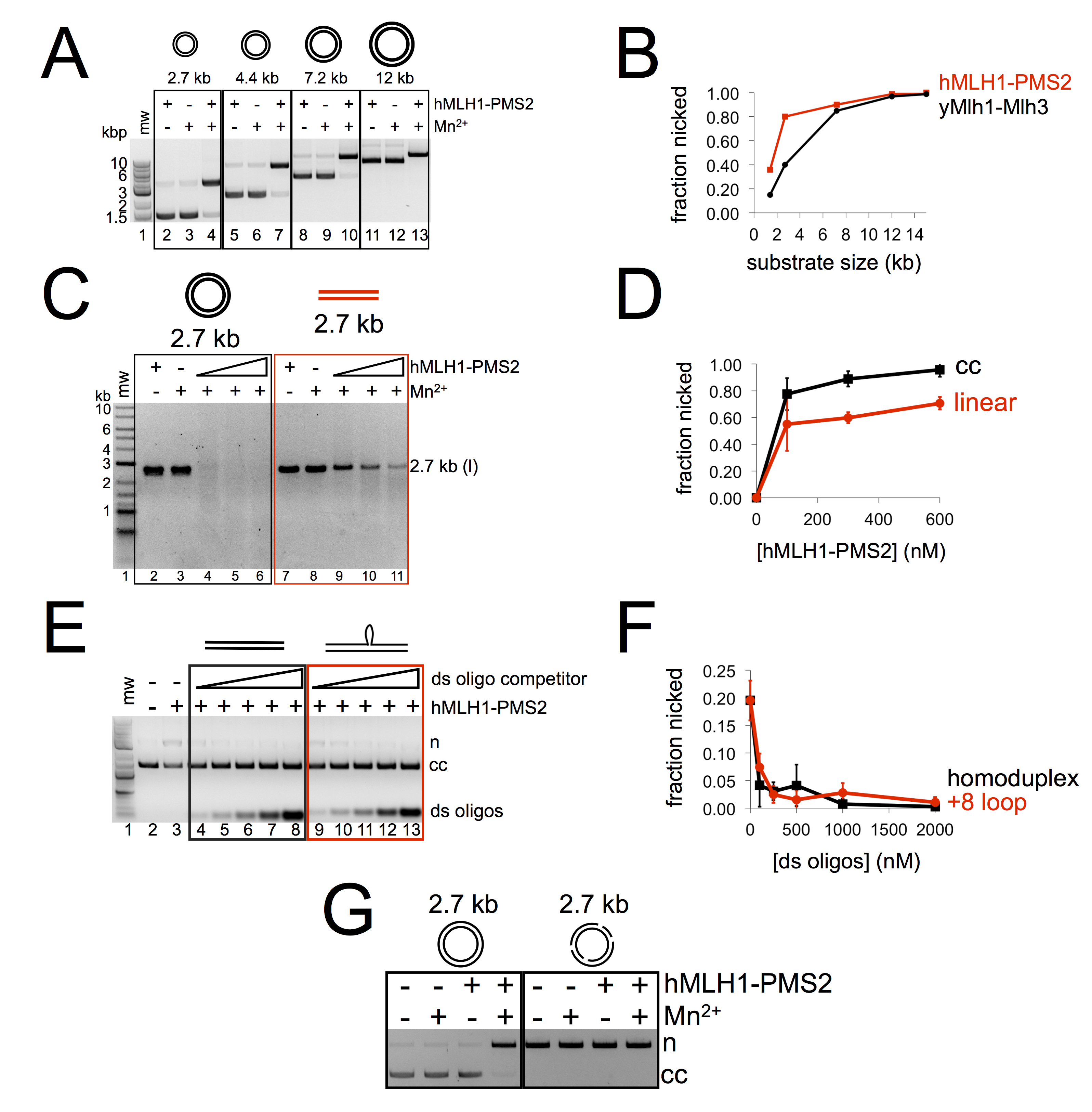

Supplement: S5 Fig — All reactions contain 1 μM yeast RFC and yeast PCNA, 0.5 mM ATP, and 1 mM Mn2+ unless otherwise indicated. (A) 15 μM total nucleotide in each reaction combined with 150 nM human MLH1-PMS2 in the presence of RFC/PCNA with ATP. Experiment is otherwise identical to that performed in Fig 2C. (B) Quantification of data in A combined with data from an identical experiment using a 1.4 kb and 15 kb circular substrate. 2.7, 7.2, and 12 kb substrates are as described in the Materials and Methods. 1.4 kb circular substrate was generated by re-ligating the ~1400 bp BssSαI fragment of pUC18. 15 kb circular substrate is plasmid pEAM58 amplified and mini-prepped from DH5α competent cells by standard methods. Reactions were combined as described in A and in Fig 2C for human MLH-PMS2 and yeast Mlh1-Mlh3. (C) Denaturing agarose analysis of human MLH1-PMS2 nicking on circular pUC18 (2.7 kb) (black) and linearized pUC18 (red). (D) Average of two separate experiments as shown in C. (E) Human MLH1-PMS2 endonuclease activity on a 2.7 kb circular DNA substrate is inhibited by pre-incubating human MLH1-PMS2 with oligonucleotide substrates. 50 nM human MLH1-PMS2 was pre-incubated with increasing amounts of ~50 bp double stranded oligonucleotide substrates for 15 minutes at 30°C (0–2000 nM), either homoduplex or substrate with a +8 loop. After the pre-incubation step, reactions were challenged with 3.6 nM 2.7 kb circular substrate and incubated by conditions described for endonuclease assays in the Materials and Methods and analyzed by agarose gel. (F) Average of quantification for four separate experiments from E, errors bars represent standard deviation. (G) hMLH1-PMS2 (300 nM) does not create linear product on 2.7 kb closed or nicked circular substrate. Reaction was performed as described for Fig 8A and 8B. (TIFF) [file pbio.2001164.s005.tiff]

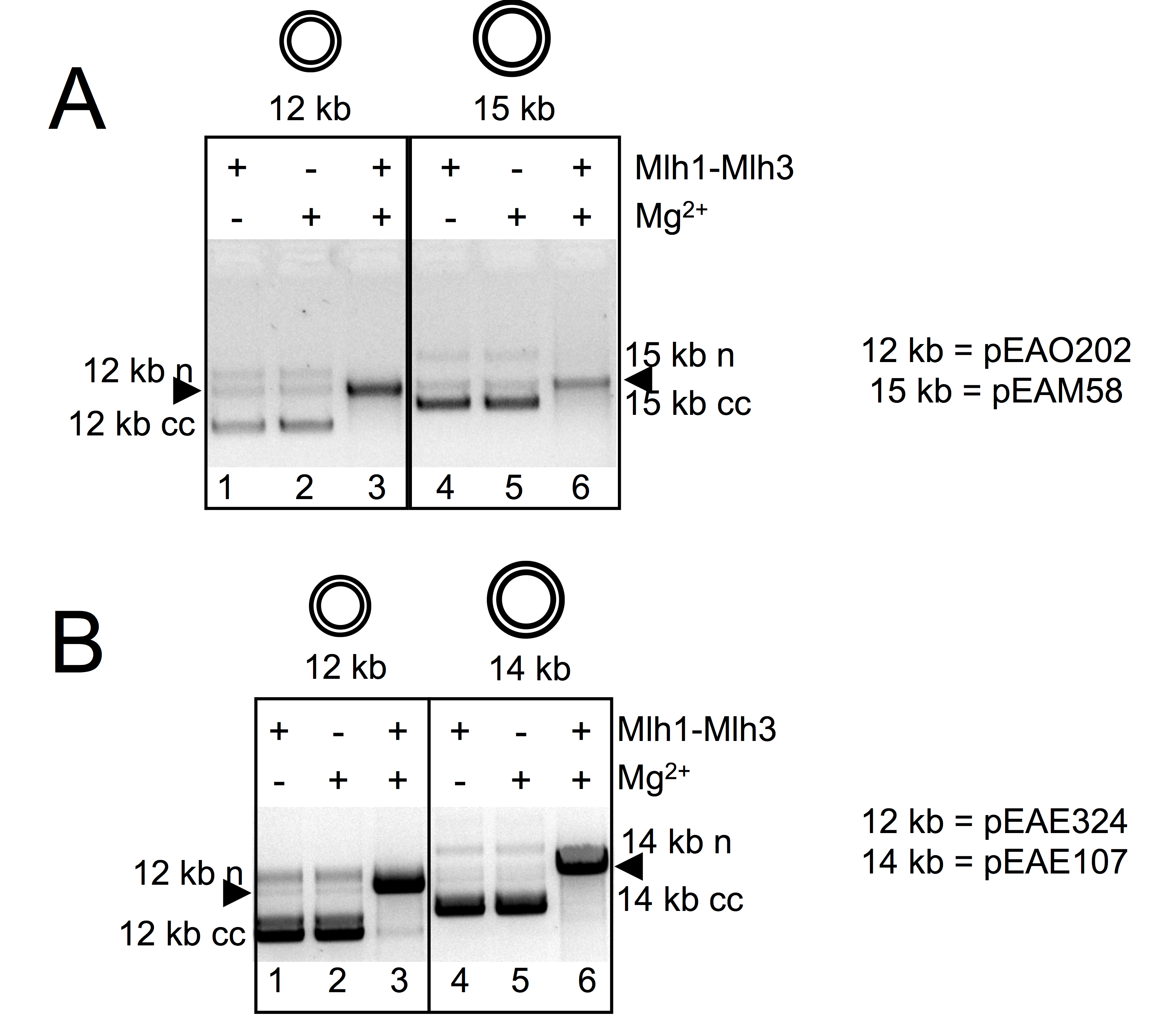

Supplement: S6 Fig — (A-B) Experiment performed identical to that in Fig 2C using the indicated sized plasmids as substrates. Sequences of 12 kb plasmids differ from that used in Figs 2C and 7. (TIFF) [file pbio.2001164.s006.tiff]

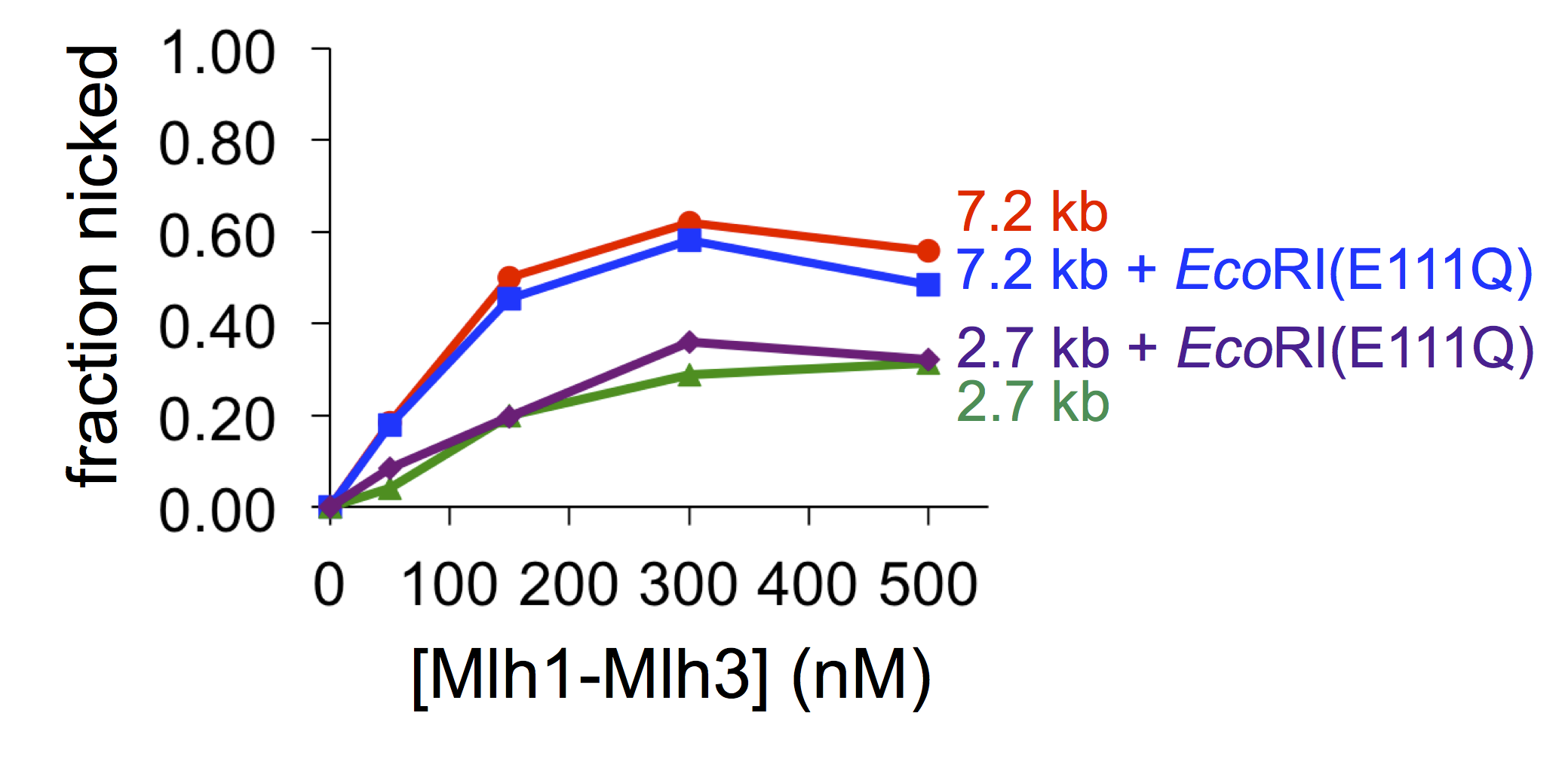

Supplement: S7 Fig — EcoRI(E111Q) is a variant of EcoRI that binds to, but does not cleave, the EcoRI recognition sequence [76]. In a 5 μL reaction, 3.2 μM of this variant was combined with 40 μM (concentration of nucleotide) DNA substrate (either 2.7 kb closed circular or 7.2 kb closed circular; each of which have one EcoRI site) in the Mlh1-Mlh3 endonuclease reaction buffer and incubated for 30 min at 37°C. Binding was confirmed by gel shift. EcoRI(E111Q)-bound substrate or substrate without EcoRI(E111Q) was then combined in an endonuclease reaction (15 μM final nucleotide concentration) with increasing amounts of Mlh1-Mlh3. The reaction was combined, allowed to proceed, and stopped as described in the Materials and Methods. Reactions were analyzed by agarose gel and quantified. (TIFF) [file pbio.2001164.s007.tiff]
