# Supplementary material for: The mismatch repair and meiotic recombination endonuclease Mlh1-Mlh3 is activated by polymer formation and can cleave DNA substrates in trans
Source: PLoS Biol. 2017 Apr 28;15(4):e2001164. doi: 10.1371/journal.pbio.2001164 (PMC5409509; doi:10.1371/journal.pbio.2001164)
Supplement: S1 Table — (DOCX) [file pbio.2001164.s008.docx]

**S1 Table. Oligonucleotides used in this study.**

| Oligonucleotide | Sequence (5’ to 3’) |
| --- | --- |
| AO3142 | GGGTCAACGTGGGCAAAGATGTCCTAGCAAGTCAGAATTCGGTAGCGTG |
| AO3144 | ACAGCTACCGAATTCTGACTTGCTAGGACATCTTTGCCCACGTTGACCC |
| AO3143 | CACGCTACCGAATTCTGACTTGCTAGGTGTGTGTGACATCTTTGCCCACGTTGACCC |
| AO3147 (X26-1) | GCGCTACCAGTGATCACCAATGGATTGCTAGGACATCTTTGCCCACCTGCAGGTTCACCC |
| AO3148 (X26-2) | GGGTGAACCTGCAGGTGGGCAAAGATGTCCTAGCAATCCATTGTCTATGACGTCAAGCTC |
| AO3149 (X26-3) | GAGCTTGACGTCATAGACAATGGATTGCTAGGACATCTTTGCCGTCTTGTCAATATCGGC |
| AO3150 (X26-3) | GCCGATATTGACAAGACGGCAAAGATGTCCTAGCAATCCATTGGTGATCACTGGTAGCGC |
| AO3266 | GGGTTTTCCCAGTCACGACGT |
| AO3267 | GGGTTTTCCCTGTGTGTGAGTCACGACGT |
| AO3346 | CGCCAGGGTTTACGACGTTGT |
| AO3516 | AGCTGATACCGCTCGCCGCAGCCGAACGAC |
| AO3518 | CTGCATTAATGAATCGGCCAACGCGCGGGG |
| AO3535 | ATGCGGTGTGAAATACCGCACAGATGCGTAAGGAGAAAATACCGCATCAGGCGCTCTTCC |
| BIO_M13mp18 | GGGTTT/iBiodT/CCCAGTCACGACGT  (**Note:** /iBiodT/ is an internal biotin dT modification from IDT) |
